# Telomeric DNA breaks in human induced pluripotent stem cells trigger ATR-mediated arrest and telomerase-independent telomere damage repair

**DOI:** 10.1093/jmcb/mjad058

**Published:** 2023-09-28

**Authors:** Katrina N Estep, John W Tobias, Rafael J Fernandez, Brinley M Beveridge, F Brad Johnson

**Affiliations:** Department of Pathology and Laboratory Medicine, University of Pennsylvania, Philadelphia, PA 19104, USA; Quantiative Biosciences, Merck & Co., Inc., West Point, PA 19486, USA; Penn Genomic Analysis Core, University of Pennsylvania, Philadelphia, PA 19104, USA; Department of Pathology and Laboratory Medicine, University of Pennsylvania, Philadelphia, PA 19104, USA; Department of Pathology and Laboratory Medicine, University of Pennsylvania, Philadelphia, PA 19104, USA; Department of Pathology and Laboratory Medicine, University of Pennsylvania, Philadelphia, PA 19104, USA

**Keywords:** telomeres, telomerase, alternative lengthening of telomeres, pluripotent stem cells, DNA damage, double-strand breaks

## Abstract

Although mechanisms of telomere protection are well-defined in differentiated cells, how stem cells sense and respond to telomere dysfunction, in particular telomeric double-strand breaks (DSBs), is poorly characterized. Here, we report the DNA damage signaling, cell cycle, and transcriptome changes in human induced pluripotent stem cells (iPSCs) in response to telomere-internal DSBs. We engineer human iPSCs with an inducible TRF1-FokI fusion protein to acutely induce DSBs at telomeres. Using this model, we demonstrate that TRF1-FokI DSBs activate an ATR-dependent DNA damage response, which leads to p53-independent cell cycle arrest in G2. Using CRISPR–Cas9 to cripple the catalytic domain of telomerase reverse transcriptase, we show that telomerase is largely dispensable for survival and lengthening of TRF1-FokI-cleaved telomeres, which instead are effectively repaired by robust homologous recombination (HR). In contrast to HR-based telomere maintenance in mouse embryonic stem cells, where HR causes ZSCAN4-dependent extension of telomeres beyond their initial lengths, HR-based repair of telomeric breaks is sufficient to maintain iPSC telomeres at a normal length, which is compatible with sustained survival of the cells over several days of TRF1-FokI induction. Our findings suggest a previously unappreciated role for HR in telomere maintenance in telomerase-positive iPSCs and reveal distinct iPSC-specific responses to targeted telomeric DNA damage.

## Introduction

Telomeres, the protective structures that shield against activation of a DNA damage response (DDR) at chromosome ends, are made up of tandem TTAGGG repeats that are bound by protective shelterin proteins and lengthened by the enzyme telomerase in order to maintain their function (Greider and Blackburn, 1985; de Lange, 2005; Shay and Wright, 2019). In dividing cells, telomere DNA shortens with each round of replication (Olovnikov, 1971; Watson, 1972), until telomeres lose sufficient TTAGGG repeats to support shelterin binding and become dysfunctional. Critical telomere erosion in differentiated cells activates a DDR that is largely dependent on the ataxia telangiectasia-mutated (ATM) kinase and the downstream activation of the p53 pathway, which promote apoptosis or senescence to halt cellular proliferation (d'Adda di Fagagna et al., 2003; Shay and Wright, 2019).

However, despite an extensive body of literature on the responses of differentiated cells and cancer cells to telomere dysfunction, the telomere capping mechanisms and cellular responses to telomere deprotection of pluripotent stem cells (PSCs) remain poorly understood. This is of particular interest considering that pathologies associated with telomere dysfunction, like those observed in telomere biology disorders, manifest in high-turnover tissues and are linked to stem cell failure (Lee et al., 1998; Hao et al., 2005; Armanios et al., 2009; Nag, 2020; Armanios, 2022). It was recently demonstrated that mechanisms for normal chromosome end protection are fundamentally different in PSCs and their differentiated counterparts. Notably, Trf2, the most critical component of the shelterin complex for proper telomere protection in differentiated cells (van Steensel et al., 1998), is completely dispensable in mouse embryonic stem cells (mESCs) (Markiewicz-Potoczny et al., 2021; Ruis et al., 2021). Furthermore, critical telomere attrition arising from deletion of human telomerase reverse transcriptase (*hTERT*), the catalytic component of telomerase, has been shown to activate a uniquely ataxia telangiectasia-related (ATR)-dependent DDR in human induced PSCs (iPSCs) that differs from the ATM-dependent response arising in more differentiated cells with critically short telomeres (Herbig et al., 2004; Vessoni et al., 2021). These observations support the concept of a PSC-specific reaction to telomere dysfunction and necessitate additional studies to determine how such cells sense and respond to other forms of telomeric DNA damage.

Here, we address how human iPSCs respond to DNA double-strand breaks (DSBs) within telomere repeats by engineering a novel human iPSC line harboring an inducible TRF1-FokI allele to acutely induce DSBs within telomeres. Telomeric DSBs activate an ATR-dependent DDR in iPSCs, cause iPSCs to undergo cell cycle arrest in G2, and maintain iPSC telomere lengths by telomerase-independent telomere recombination.

## Results

### A novel iPSC model of inducible telomere dysfunction

**Figure 1:**
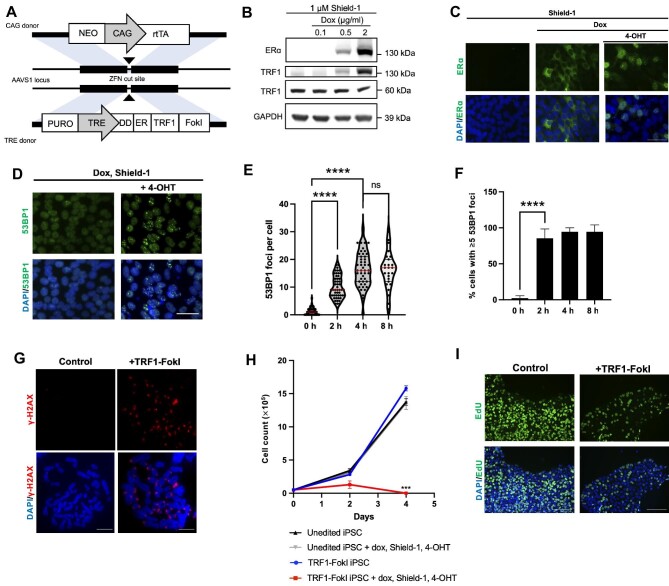
Generation of targeted dox-inducible TRF1-FokI iPSC model. (**A**) Strategy for targeted integration of CAG-rtTA and DD-ER-TRF1-FokI into AAVS1 safe harbor loci. (**B**) Western blot analysis confirming the expression and stabilization of TRF1-FokI fusion protein (130 kDa) using antibodies against ERα and TRF1. Cells were induced for 48 h with the indicated concentrations of dox and Shield-1. The level of native TRF1 (60 kDa) was unaffected. (**C**) Immunofluorescence staining confirming cytoplasmic accumulation of TRF1-FokI fusion protein after 48-h treatment with dox and Shield-1 and the internalization of TRF1-FokI into the nucleus after additional treatment with 4-OHT for 16 h. Scale bar, 50 μm. (**D**) Immunofluorescence staining for 53BP1 foci after 2-h induction of TRF1-FokI. Scale bar, 50 μm. (**E** and **F**) Quantification of 53BP1 foci as a function of time after nuclear induction of TRF1-FokI. Cells were pre-treated with dox for 48 h and induced by addition of Shield-1 and 4-OHT. A total of 50–100 nuclei were analyzed per time point across three independent experiments. *P*-values are from one-way ANOVA. ****P < 0.0001; ns, no significance. (**G**) Immunofluorescence staining for γ-H2AX in metaphase chromosomes harvested from uninduced cells (control) and cells after 4-h nuclear induction of TRF1-FokI. Scale bar, 10 μm. (**H**) Growth curve for unedited parental iPSCs and TRF1-FokI iPSCs either under normal growth conditions or induced following addition of 1 μg/ml dox, 1 μM Shield-1, and 1 μM 4-OHT. Data are representative of three independent experiments. *P*-value for Day 4 is from unpaired two-tailed *t*-test (*P* = 0.0003). (**I**) EdU staining of TRF1-FokI iPSCs uninduced (control, dox only) or following 72 h of induction with 1 μM Shield-1 and 1 μM 4-OHT. Scale bar, 100 μm. EdU was added to cultures for 30 min prior to fixation.

To engineer a human iPSC line in which telomeric DSBs can be conditionally induced, we used an established strategy for targeted integration of a doxycycline (dox)-inducible transgene expression system (Sim et al., 2016) and introduced TRE-DD-ER-TRF1-FokI and CAG-rtTA constructs into the AAVS1 safe harbor loci (Figure 1A). The TRF1-FokI construct contains a destabilization domain (DD) degron and a modified estrogen receptor alpha (ERα) to allow for the controlled stabilization (by Shield-1 ligand) and subcellular localization (by 4-hydroxytamoxifen; 4-OHT) of a TRF1-FokI fusion protein, which localizes the FokI endonuclease to telomere repeats upon entry into the nucleus (Cho et al., 2014; Dilley et al., 2016; Supplementary Figure S1A). We confirmed that our inducible TRF1-FokI protein was expressed upon addition of dox and Shield-1 ligand (Figure 1B). Immunofluorescence microscopy confirmed a lack of detectable protein expression in the absence of dox and the internalization of TRF1-FokI into the nucleus upon addition of 4-OHT (Figure 1C). The TRF1-FokI iPSC line exhibited a normal karyotype (Supplementary Figure S1D) and retained expression of the transcription factor Nanog, a marker of pluripotency (Supplementary Figure S1E).

Previous studies in differentiated cells demonstrated that TRF1-FokI and 53BP1 colocalize at telomeres, indicating a DDR to telomeric breaks (Cho et al., 2014; Dilley et al., 2016; Doksani  and de Lange, 2016). In our TRF1-FokI iPSCs, a time-dependent accumulation of 53BP1 foci was observed following nuclear induction of TRF1-FokI (Figure 1D), with a significant increase in the average number of 53BP1 foci per cell at 2 h and a peak at 4 h post-induction, when nearly all cells exhibited >5 foci (Figure 1E and F). To verify that the damage was restricted to telomeres, we arrested cells in metaphase and performed immunofluorescence staining for the DNA damage marker γ-H2AX. As expected, cells induced to express nuclear TRF1-FokI for 4 h exhibited metaphase chromosomes with γ-H2AX foci present at chromosome ends, i.e. metaphase telomere dysfunction-induced foci (Figure 1G; Supplementary Figure S2). Non-telomeric γ-H2AX foci occurred with comparable frequency under uninduced and induced conditions (Supplementary Figure S2D), confirming that TRF1-FokI selectively cleaves telomeric DNA rather than cutting promiscuously throughout the genome.

To gauge the kinetics of iPSC responses to TRF1-FokI, we monitored cell counts and DNA synthesis as indicated by ethynyl-2-deoxyuridine (EdU) incorporation. TRF1-FokI led to a severe growth defect after 2 days and near complete cell losses after 4 days, as well as diminished EdU incorporation at 72 h (Figure 1H and I). The lack of any surviving cells beyond 96 h indicates that most or all cells accumulated toxic levels of TRF1-FokI. Thus, our model allows us to swiftly express TRF1-FokI to selectively induce robust telomeric DNA damage in iPSCs.

### TRF1-FokI DSBs arrest iPSCs in G2 through an ATR-dependent mechanism

**Figure 2:**
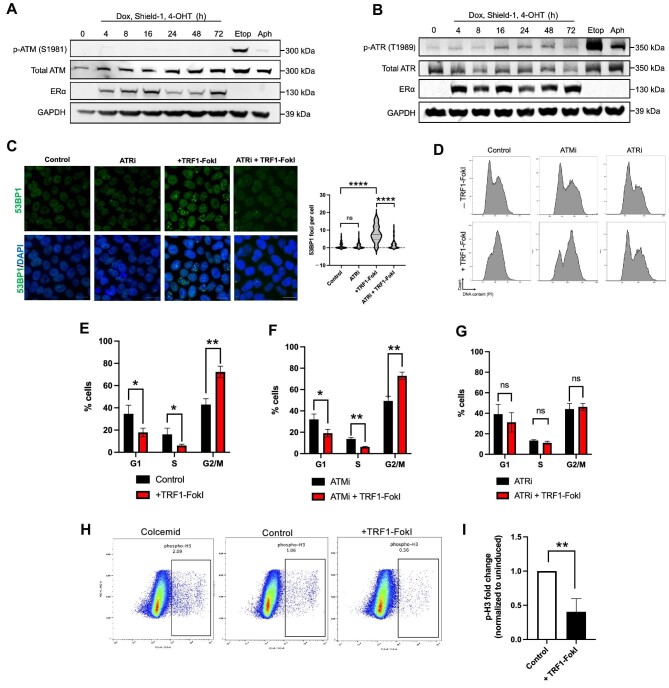
TRF1-FokI DSBs activate ATR signaling to arrest iPSCs in G2. (**A** and **B**) Western blot analysis of ATM and ATR signaling following induction of TRF1-FokI for the indicated duration or treatment with high-dose etoposide (10 μM) or aphidicolin (10 μM) for 2 h. (**C**) Immunofluorescence staining measuring the effect of ATRi on 53BP1 foci accumulation following 4 h of TRF1-FokI induction. Cells were co-treated during TRF1-FokI induction with the ATR inhibitor VE821. Scale bar, 20 μm. *P*-values are from one-way ANOVA. ****P < 0.0001; ns, no significance. (**D**–**G**) Effects of ATMi and ATRi on cell cycle following 8 h of TRF1-FokI induction. Cells were co-treated during TRF1-FokI induction with the ATM inhibitor KU-55933 (10 μM) or the ATR inhibitor VE821 (1 μM). DNA content was measured by flow cytometry for propidium iodide (PI) and quantified from three independent experiments. *P*-values are from unpaired two-tailed *t*-tests. (**E**) *P* = 0.028, *P* = 0.039, and *P* = 0.002, respectively. (**F**) *P* = 0.022, *P* = 0.001, and *P* = 0.002, respectively. (**G**) *P* = 0.364, *P* = 0.095, and *P* = 0.616, respectively. (**H** and **I**) p-H3 levels in control and TRF1-FokI-induced cells as analyzed by flow cytometry (**H**) and quantified from three independent experiments (**I**). Positive control cells were treated with 0.4 μg/ml colcemid for 1 h to arrest cells in mitosis. *P*-value is from unpaired two-tailed *t*-test (*P* = 0.006).

Previous work has indicated that human pluripotent cells differ from their isogenic differentiated counterparts in their responses to progressive telomere erosion caused by genetic inactivation of *hTERT* (Vessoni et al., 2021). In particular, iPSCs experiencing critical telomere shortening activate a DDR largely dependent on the ATR kinase, rather than the ATM kinase that is more prominent in differentiated cells (Doksani and de Lange, 2016). We were thus curious which pathway governs responses to acute telomeric DSBs in iPSCs. Western blot analysis across 72 h of TRF1-FokI induction showed a notable increase in phosphorylated ATR but not phosphorylated ATM (Figure 2A and B). Co-treatment with the ATR inhibitor VE821 during TRF1-FokI induction led to a complete rescue of nuclear 53BP1 foci (Figure 2C), confirming a functional role for ATR in TRF1-FokI damage signaling. Flow cytometry revealed a time-dependent G2/M accumulation in the induced TRF1-FokI iPSCs, with ∼50% of cells arrested in G2/M after 4 h of induction and >70% after 8 h (Figure 2D; Supplementary Figure S3A and B). Total protein levels of CHK1 and CHK2 kinases, downstream targets of ATR, increased after induction (Supplementary Figure S3C), presumably as a consequence of G2/M accumulation, but CHK1 phosphorylation level did not increase (Supplementary  Figure S3D), indicating that CHK1 does not mediate the effects of ATR. Co-treatment with VE821 but not the ATM inhibitor KU-55933 or the CHK1 inhibitor Prexasertib resulted in a complete rescue of G2/M arrest at 8 h of TRF1-FokI induction (Figure 2D–G; Supplementary Figure S3E), confirming a key role for ATR but not ATM or CHK1. Notably, unedited parental cells (i.e. lacking the TRF1-FokI transgene) treated with the induction medium (i.e. dox, Shield-1, and 4-OHT) did not exhibit any change in cell cycle distribution (Supplementary Figure S3F and G), indicating the arrest dependent on TRF1-FokI.

Telomere erosion in iPSCs was previously shown to trigger mitotic instability, resulting in cell death during mitosis (Vessoni et al., 2021). To examine whether iPSCs experiencing experimental telomeric breaks similarly undergo mitotic arrest, we performed intracellular flow cytometry for phosphorylated histone H3 (p-H3) in TRF1-FokI iPSCs. Surprisingly, cells induced for 8 h exhibited on average a 2-fold decrease in the percentage of p-H3-positive cells compared to control cells, inconsistent with a mitotic arrest phenotype (Figure 2H and I). These data collectively led us to conclude that TRF1-FokI-induced telomeric DSBs arrest iPSCs in G2 by activating an ATR-dependent DDR.

### RNA-sequencing analysis across a time course of TRF1-FokI induction

**Figure 3:**
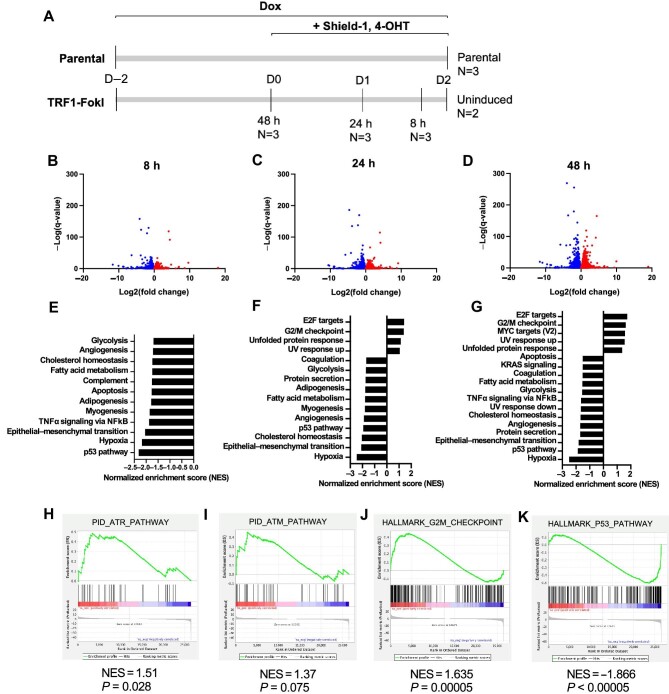
RNA-seq reveals time-dependent gene expression changes in TRF1-FokI-induced iPSCs. (**A**) Experimental setup for RNA-seq experiment. TRF1-FokI cells and unedited parental cells were plated at the same density 48 h prior to the induction in the medium containing 1 μg/ml dox. Cells were then induced for the indicated duration with 1 μg/ml Shield-1 and 1 μg/ml 4-OHT. Multiple replicates per condition were collected together at the experiment endpoint on Day 2. Parental cells treated with dox, Shield-1, and 4-OHT throughout the experiment served as a control to account for gene expression changes driven by exposure to Shield-1 and 4-OHT. (**B**–**D**) Volcano plots showing genes significantly (<0.05) upregulated (red) or downregulated (blue) in TRF1-FokI cells induced for 8, 24, and 48 h compared to unedited parental cells. (**E**–**G**) GSEA hallmark gene sets significantly (<0.05) upregulated or downregulated in TRF1-FokI cells induced for 8, 24, and 48 h compared to unedited parental cells. (**H** and **I**) GSEA indicates significant upregulation of ATR pathway (**H**) but not ATM pathway (**I**) gene set at 8 h of induction compared to unedited parental cells. (**J** and **K**) GSEA indicates significant upregulation of G2/M checkpoint (**J**) and significant downregulation of p53 pathway (**K**) gene sets at 48 h of TRF1-FokI induction compared to unedited parental cells.

Given that pluripotent mESCs lacking the shelterin Trf2 activate specialized gene expression programs required for telomere maintenance compared to their isogenic differentiated counterparts (Markiewicz-Potoczny et al., 2021), we next performed bulk RNA sequencing (RNA-seq) to assess global gene expression changes in TRF1-FokI iPSCs through a time course of TRF1-FokI induction. TRF1-FokI iPSCs after 8, 24, and 48 h of TRF1-FokI induction were either compared to uninduced cells (treated for 48 h with dox only) or compared to unedited parental iPSCs treated for 48 h with dox, Shield-1, and 4-OHT (Figure 3A). A sizeable proportion of significantly differentially expressed genes at 48 h of induction were also significantly differentially expressed in parental cells compared to uninduced cells (Supplementary Figure S4), which were driven by exposure to 4-OHT and Shield-1 rather than TRF1-FokI expression. Thus, parental cells treated with the induction medium were more appropriate to be a control group. Consistently, gene set enrichment analysis (GSEA) of RNA-seq data demonstrated robust activation of ATR but not ATM signaling (Figure 3H and I) and G2/M checkpoint-associated genes (Figure 3J) in TRF1-FokI iPSCs.

### Long-term TRF1-FokI induction leads to non-apoptotic death of iPSCs

Telomere-internal DSBs have been shown to induce differential apoptotic or senescence responses depending on the cell type (Fumagalli et al., 2012; Hewitt et al., 2012; Sun et al., 2015; Yu et al., 2015; Anderson et al., 2019). To assess effects of persistent telomere-internal breaks in iPSCs, we performed a 72-h time course of TRF1-FokI induction and examined cell viability, apoptosis, and senescence. TRF1-FokI iPSCs showed a time-dependent decline in viability, with complete cell loss during 72–96 h (Figure 1H). The cells remained largely negative for senescence-associated β-galactosidase (SA β-gal) at all time points of induction (<4%, Supplementary Figure S5A), compared to IMR90 fibroblasts passaged to the point of replicative senescence (>35%). RNA-seq analysis also demonstrated that both p16 and p21 mRNA levels decreased after induction (Supplementary Figure S5B and C). These observations agree with previous reports that iPSCs do not undergo senescence in response to telomere dysfunction (Vessoni et al., 2021). iPSCs are primed to undergo apoptosis in response to genotoxic stress (Momcilovic et al., 2010; Shimada et al., 2019; Sjakste and Riekstiņa, 2021). There was a progressive increase in annexin V positivity, although this affected only a minority of cells (Supplementary Figure S5D). Furthermore, there was no detectable cleaved caspase 3 protein after 72 h of induction (Supplementary Figure S6A), and treatment with the pan-caspase inhibitor Z-VAD-FMK failed to rescue cell growth across the induction period (Supplementary  Figure S5E), suggesting a caspase 3-independent death mechanism. Additionally, GSEA hallmarks from RNA-seq showed a significant downregulation of the apoptosis pathway at all three time points (Figure 3E–G). Taken together, these observations suggest that TRF1-FokI cells die by a non-apoptotic mechanism.

### Loss of p53 abrogates cell death without rescuing G2 arrest

**Figure 4:**
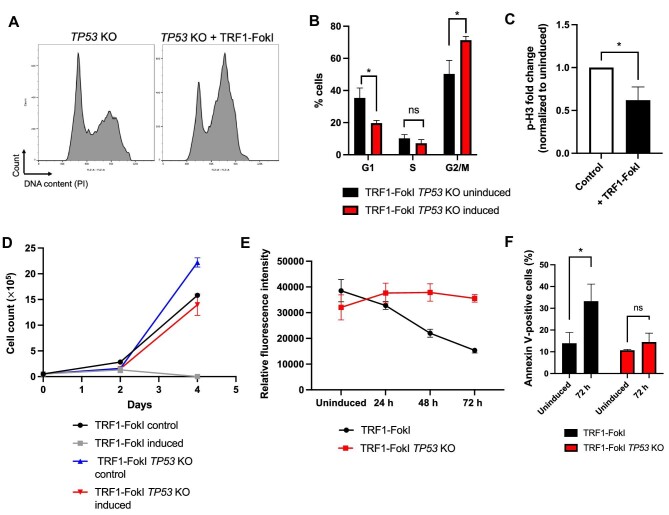
p53 loss attenuates cell death but not G2 arrest following telomeric DSBs in iPSCs. (**A** and **B**) Cell cycle analysis of control (dox only) and 8-h TRF1-FokI-induced *TP53* KO cells. DNA content was measured by flow cytometry and quantified from three independent experiments. *P*-values are from unpaired two-tailed *t*-tests (*P* = 0.014, *P* = 0.179, and *P* = 0.015, respectively). (**C**) Quantification of p-H3 levels measured by flow cytometry in uninduced (control) and TRF1-FokI-induced *TP53* KO cells. *P*-value is from unpaired two-tailed *t*-test (*P* = 0.013). (**D**) Growth curve for TRF1-FokI iPSCs (with wild-type *TP53*) and TRF1-FokI *TP53* KO iPSCs either under normal growth conditions (control) or induced following addition of 1 μg/ml dox, 1 μM Shield-1, and 1 μM 4-OHT. Data are representative of three independent experiments. (**E**) Fluorometric cell viability assay measuring total cell viability of parental TRF1-FokI or TRF1-FokI *TP53* KO cells over 72 h of TRF1-FokI induction. Data represent the mean and SD from triplicate samples measured per time point per cell line. (**F**) Quantification of annexin V positivity in parental TRF1-FokI or TRF1-FokI *TP53* KO cells before induction and after 72 h of induction as analyzed by flow cytometry and quantified from three independent experiments. *P*-values are from unpaired two-tailed *t*-tests (*P* = 0.022 and *P* = 0.06, respectively).

To gain a better understanding of DDR signaling in TRF1-FokI iPSCs downstream of ATR, we turned our attention to p53, a key mediator of the DDR. We were surprised to see p53 pathway genes among the most strongly downregulated gene sets at all three time points of TRF1-FokI induction (Figure 3E–G and K). Western blot analysis confirmed that p53 protein levels remained constant throughout 72 h of TRF1-FokI induction, in contrast to cells treated with high-dose etoposide, in which p53 levels increased by ∼2-fold within 2 h (Supplementary Figure S6A). This observation prompted us to ask whether p53 is required for cell cycle arrest following induction of telomeric DSBs in iPSCs. To test this, we ablated *TP53* using clustered regularly interspaced short palindromic repeats (CRISPR)–Cas9 in TRF1-FokI iPSCs (Supplementary Figure S6B and C). The resulting cell line (TRF1-FokI *TP53* KO) exhibited normal karyotype and was resistant to the p53-stabilizing drug Nutlin-3 (Supplementary Figure S6D and E). Cell cycle analysis revealed that loss of p53 had no effect on G2 accumulation after 8 h of TRF1-FokI induction (Figure 4A–C), consistent with previous reports that p53 is not required for cell cycle checkpoint activation in iPSCs with telomeres uncapped by progressive erosion (Vessoni et al., 2021). p53 has been reported to regulate cell death through mechanisms distinct from its role in cell cycle checkpoint activation (Chen, 2016). Consistent with this, we observed that TRF1-FokI *TP53* KO cells were protected against elevated annexin V and cell death after persistent TRF1-FokI induction, with growth kinetics similar to those observed for uninduced parental TRF1-FokI control cells over 72 h (Figure 4D–F). These results indicate that in iPSCs, p53 is not involved in cell cycle checkpoint activation resulting from telomeric DSBs but does regulate cell death following prolonged telomeric DNA damage.

### Human iPSCs do not activate a totipotent-like two-cell-stage transcriptional program in response to telomeric DSBs

mESCs are capable of adopting a two-cell (2C)-like gene expression state characterized by high expression of the zinc finger and SCAN domain containing 4 (*Zscan4*) gene cluster, which is critical for telomere protection in the absence of the shelterin Trf2 (Markiewicz-Potoczny et al., 2021). To delineate differences between mouse and human pluripotent cells, as well as the nature of responses to acute telomeric DSBs vs. Trf2 deletion, we examined whether 2C-like genes were significantly altered in our system following TRF1-FokI expression. RNA-seq showed that *ZSCAN4* transcripts were not enriched at any of the induced time points (Supplementary Figure S7A), and neither were transcripts mapping to the transcription factor *DUX4* (not detected), which in mouse drives expression of *Zscan4* and regulates the 2C state in mESCs (Hendrickson et al., 2017; Vuoristo et al., 2022). Additionally, GSEA confirmed that ZSCAN4 transcriptional targets were not enriched at any of the three time points (Supplementary Figure S7B). We next asked whether human orthologues of 13 other murine 2C genes previously shown to be upregulated following *Trf2* deletion in mESCs were upregulated or not in our system. We detected 6 out of 7 such 2C genes that have conserved human orthologues by RNA-seq; however, only two of them were significantly upregulated at 48 h of induction (Supplementary Figure S7C). We thus conclude that human iPSCs do not activate a ZSCAN4-dependent 2C-like transcriptional state in response to targeted telomeric DSBs.

### TRF1-FokI breaks result in negligible telomere shortening and are efficiently repaired by homologous recombination

**Figure 5:**
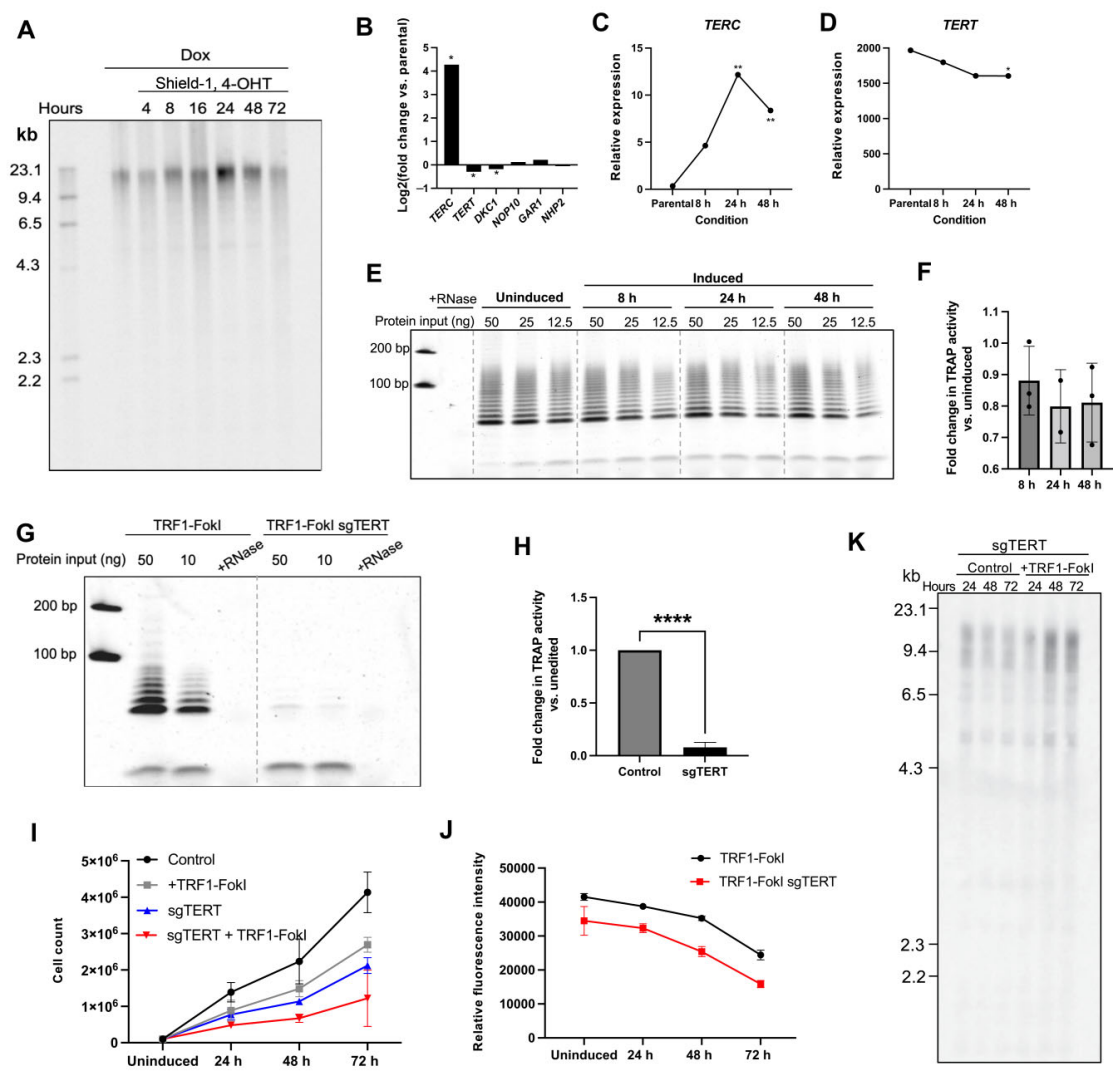
Telomere length is well-maintained following prolonged induction of TRF1-FokI. (**A**) TRF analysis of telomere lengths in cells uninduced or induced to express TRF1-FokI for the indicated duration. (**B**) mRNA expression levels of components of the telomerase holoenzyme in TRF1-FokI iPSCs after 48 h of induction compared to uninduced parental cells as measured by RNA-seq. *P < 0.05. (**C** and **D**) Trends of relative mRNA expression of *TERC* and *TERT* at the indicated time points as measured by RNA-seq. **P < 0.01, *P < 0.05. (**E**) The TRAP assay measuring telomerase enzymatic activity in TRF1-FokI iPSCs using 2-fold extract dilutions. (**F**) Quantification of fold change in TRAP activity in 8-, 24-, and 48-h induced TRF1-FokI iPSCs compared to uninduced cells. (**G**) TRAP assay measuring telomerase enzymatic activity in uninduced TRF1-FokI iPSCs (with wild-type *hTERT*) and biallelic *hTERT* mutant TRF1-FokI iPSCs (sgTERT) using 5-fold extract dilutions. (**H**) Quantification of fold change in TRAP activity in sgTERT cells compared to parental TRF1-FokI iPSCs. Data represent mean and SD from two independent experiments. ****P < 0.0001. (**I**) Growth curve for TRF1-FokI (with wild-type *hTERT*) and sgTERT iPSCs. All cells were treated with 1 μg/ml dox throughout the experiment. Cells were either mock-induced (control, dox only) or induced (1 μg/ml dox, 1 μM Shield-1, and 1 μM 4-OHT) for the indicated duration, and then total cells were counted at each time point. (**J**) Fluorometric cell viability assay measuring total cell viability of parental TRF1-FokI or sgTERT cells over 72 h of TRF1-FokI induction. Data represent the mean and SD from 6 samples measured across two plates per time point per cell line. (**K**) TRF analysis of telomere lengths in sgTERT iPSCs either mock-induced (control) or induced to express TRF1-FokI (+TRF1-FokI) for the indicated duration.

We next asked how persistent expression of TRF1-FokI impacts telomere length. We hypothesized that prolonged induction of TRF1-FokI would lead to a time-dependent decrease in bulk telomere length as telomeres are cleaved, as has been shown to be the case for mouse embryonic fibroblasts (Doksani and de Lange, 2016). Surprisingly, persistent expression of TRF1-FokI in iPSCs resulted in negligible telomere shortening as analyzed by terminal restriction fragment (TRF) analysis using both standard and pulse field gel electrophoresis (Figure 5A; Supplementary Figure S8). This was unexpected, given that 53BP1 and γ-H2AX staining indicated widespread telomere cleavage (Figure 1). We also considered the possibility that rather than causing DSBs (as expected, but never shown directly for TRF1-FokI), the nuclease primarily induces telomere nicks, which could then be converted to DSBs when encountered by a replication fork, thus providing a setting in which such DSBs could efficiently be repaired by sister telomere homologous recombination (HR) and thus minimizing their steady-state levels (see below and Discussion). However, TRF1-FokI induction did not lead to any apparent increase in the sensitivity of telomeres to digestion with S1 nuclease, which can convert nicks to DSBs (Supplementary Figure S9).

Compared to differentiated cells, iPSCs have long telomeres maintained by high levels of *hTERT* and robust telomerase activity (Huang et al., 2014). We therefore hypothesized that the lack of telomere shortening was a result of efficient extension of cleaved telomeres by telomerase with kinetics comparable to the rate of cleavage. We first asked whether we could detect elevated telomerase activity in response to TRF1-FokI breaks, as other DSB-inducing agents (including ionizing radiation and etoposide) have been shown to boost telomerase expression and enzymatic activity in various cellular contexts (Sato et al., 2001; Sishc et al., 2015). Our RNA-seq data was consistent with this idea, as the limiting telomerase RNA component (*TERC*), but not *TERT*, showed significantly increased expression at 24 and 48 h (Figure 5B–D). However, a functional test for telomerase enzymatic activity by telomerase repeat amplification protocol (TRAP) assay showed no detectable change in TRAP activity with TRF1-FokI induction (Figure 5E and F), indicating that TRF1-FokI breaks do not promote increased telomerase activity. To directly test the contribution of telomerase in maintaining telomere lengths following FokI breaks, we used CRISPR–Cas9 to target the catalytic domain of *hTERT* in TRF1-FokI iPSCs. We obtained a surviving hypomorph clone (sgTERT) with biallelic indel mutations within the reverse transcriptase domain that severely compromised telomerase activity (Figure 5G and H; Supplementary Figure S10). sgTERT cells displayed reduced growth kinetics at baseline compared to the parental TRF1-FokI line (Figure 5I), but taking this into account, the rate of impaired growth following induction of TRF1-FokI was no more severe for sgTERT cells than it was for cells with wild-type *TERT* (Figure 5J). Furthermore, although TRF analysis of sgTERT cells revealed obvious telomere shortening during the ∼25 divisions since its generation (cf. Figure 5K vs. A), additional telomere shortening was not apparent over 3 days of TRF1-FokI induction. Collectively, these data led us to conclude that telomere lengths are maintained following persistent TRF1-FokI breaks through a largely telomerase-independent mechanism.

**Figure 6:**
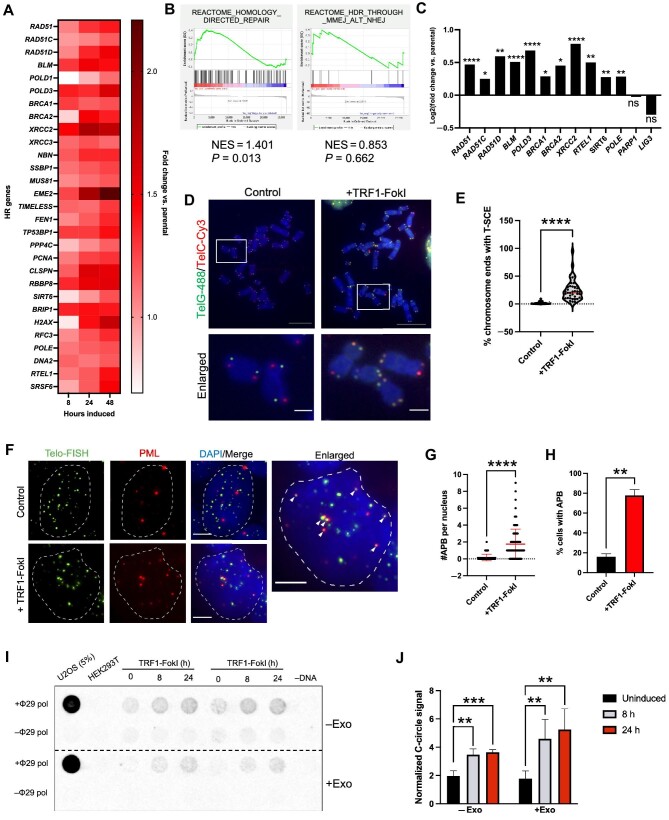
TRF1-FokI DSBs activate HR in iPSCs. (**A**) Heat map depicting fold change in relative expression normalized to unedited parental cells of HR-related genes throughout the 48-h induction period. (**B**) GSEA indicating a significant enrichment of the homology-direct repair reactome gene signature, but not the MMEJ/alt-NHEJ signature, at 48 h of TRF1-FokI induction. (**C**) Significant upregulation of genes associated with HR at 48 h of TRF1-FokI induction, measured by log2(fold change vs. parental). *P < 0.05, **P < 0.01, ****P < 0.0001; ns, no significance. (**D**) CO-FISH performed on metaphase spreads harvested from uninduced (dox only) and 4-h induced TRF1-FokI iPSCs. Leading strand telomeres were hybridized with the TelG-488 probe (green) and lagging strand telomeres were hybridized with the TelC-Cy3 probe (red). Scale bar, 10 μm (top panels) or 2 μm (enlarged panels). (**E**) Quantification of T-SCE frequency as detected by CO-FISH. Data are representative of three independent experiments. ****P < 0.0001. (**F**) Combined immunofluoresence and FISH staining for telomeric DNA (green) and PML protein (red) in uninduced control and 48-h induced TRF1-FokI iPSCs. Arrows indicate representative APBs. Scale bar, 5 μm. (**G** and **H**) Quantification of discrete number of APBs per nucleus (**G**) and the fraction of cells containing one or more APBs (**H**) in uninduced control and 48-h induced TRF1-FokI iPSCs. Data are representative of at least 100 nuclei across three independent experiments. ****P < 0.0001 (**G**) and *P* = 0.006 (**H**) are from unpaired two-tailed *t*-test. (**I** and **J**) The C-circle assay measuring C-circle abundance in uninduced, 8-h induced, and 24-h induced TRF1-FokI iPSCs. (**I**) Representative dot blot showing two biological replicates for bulk genomic DNA without (top) and with exonuclease (Exo) digestion (bottom). ALT+ U2OS and ALT– HEK293T DNAs were included as positive and negative controls, respectively. Sample-specific background is quantified from the signal generated in the absence of ϕ29 polymerase. (**J**) Quantification of relative C-circle abundance. C-circle signal for each sample is normalized to sample-specific background. Data represent average and SD from two technical replicates and two biological replicates. **P < 0.01, ***P < 0.001.

Telomere recombination is central to telomerase-independent telomere lengthening mechanisms, which includes so called alternative lengthening of telomeres (ALT). mESCs can employ ALT-like telomere lengthening (Liu et al., 2007; Zalzman et al., 2010; Varela et al., 2011), but whether such mechanisms can occur following acute telomeric breaks in human iPSCs has not been tested previously. To begin to examine this possibility, we conducted a focused analysis of RNA-seq data for major DNA repair pathways. Genes associated with homology-directed repair were generally upregulated, including *RAD51* and its paralogs *RAD51B* and *RAD51C*, as well as *BLM, POLD3, BRCA1*, and *BRCA2*, and this was corroborated by GSEA (Figure 6A–C). Importantly, there was no enrichment of genes associated with the microhomology-mediated/alternative non-homologous end joining (MMEJ/alt-NHEJ) pathway, including *LIG3* and *PARP1*, by which telomeric FokI breaks are repaired in differentiated cells (Doksani and de Lange, 2016; Figure 6B and C).

To test whether TRF1-FokI cells exhibit features of telomere recombination, we first carried out chromosome orientation fluorescence *in situ* hybridization (CO-FISH), which can be used to visually detect telomeric sister chromatid exchange events (T-SCEs) (Bailey et al., 1996, 2004). We detected a significant increase in the frequency of T-SCEs in cells induced to express TRF1-FokI for 4 h (Figure 6D and E), confirming active recombination between telomeres. Immunofluorescence staining also revealed a significant increase in the colocalization of PML bodies with TTAGGG repeats (Figure 6F–H), which indicate nuclear sites of telomere recombination (Yeager et al., 1999; Grobelny et al., 2000). Furthermore, we probed for C-rich telomeric DNA circles (C-circles) by rolling circle amplification, which are characteristic of ALT (Henson et al., 2009, 2017). C-circles were present in TRF1-FokI iPSCs, and they became more abundant with induction (Figure 6I and J). The abundance of C-circles in TRF1-FokI iPSCs was low compared to ALT+ U2OS cells, raising the question as to whether the signal might be from spurious amplification of nicked linear telomeric DNA rather than C-circles. However, the signal remained after restriction digestion and exonuclease treatment to remove linear genomic DNA, confirming that the signal was amplified entirely from circular template. Taken together, these results indicate that telomeric DSBs activate robust telomere recombination in telomerase-positive iPSCs.

## Discussion

We engineered a human iPSC line with an inducible TRF1-FokI construct to study the impact of acute telomeric DSBs on iPSC growth, survival, DNA damage signaling, and repair mechanisms. Protein and cell cycle analyses showed that such breaks activate ATR signaling to arrest cells in G2. Importantly, this finding differs from the primarily ATM-dependent response to the same form of targeted telomeric DSBs in differentiated cells (Doksani and de Lange, 2016). Since HR-based repair takes place predominantly in S and G2, it is tempting to speculate that ATR rather than ATM activation in this context serves to promote telomere recombination as a way to achieve efficient and error-free repair. This would be a particularly useful telomeric repair mechanism for PSCs, which must preserve genomic integrity in order to faithfully serve as the progenitors of an entire organism. Importantly, cell cycle arrest in this context appears to occur through a noncanonical mechanism that is independent of both CHK1 and p53, and future studies will be required to fully characterize the nature of this signaling cascade.

Our finding that iPSCs do not undergo senescence or canonical apoptosis in response to targeted telomeric DSBs is also in stark contrast to what is known to occur in other cell types. In differentiated cells, prolonged cell cycle checkpoint activation engages downstream apoptosis or senescence to halt proliferation of genetically unstable cells. While additional studies are needed to pinpoint the precise mechanism by which our TRF1-FokI iPSCs die, we hypothesize that a necroptosis-like mechanism might be at play, as it is associated with annexin V positivity but not caspase activation (Shlomovitz et al., 2019) and can occur independently of p53 (Li et al., 2015). Necroptosis has been tightly linked to upregulation of the unfolded protein response (UPR) pathway (Saveljeva et al., 2015; Liang et al., 2021; McGrath et al., 2021), which our RNA-seq detected was significantly elevated at later time points of TRF1-FokI induction (Figure 3). As an aside, we and others have previously observed upregulation of the UPR in the setting of telomere dysfunction in other cellular contexts (Hosoi et al., 2016; Fernandez et al., 2022), suggesting that telomere damage may be a general driver of ER stress.

It is noteworthy that we were unable to obtain iPSCs that are fully null for *hTERT*. This differs from the case for murine ESCs and iPSCs and presumably reflects the well-established greater susceptibility of humans to telomerase deficiency. For instance, first-generation mice null for telomerase activity are nearly normal (Blasco et al., 1997; Strong et al., 2011), whereas people with hypomorphic alleles in telomerase components can exhibit severe defects in childhood (Savage and Bertuch, 2010; Tummala et al., 2022). It also appears to contrast with a recent report that *hTERT* could be deleted fully in human iPSCs (Vessoni et al., 2021), but these cells contained a dox-inducible *hTERT* transgene which was expressed during generation of the endogenous *hTERT* knockout and thus presumably maintained viability. An essential role for telomerase in human iPSCs may reflect one or more of the apparent extra-telomeric roles for telomerase (Martínez and Blasco, 2011; Thompson and Wong, 2020), consistent with the poor growth of our hypomorphic clone even at relatively long telomere lengths. It is also remarkable that telomeres shortened with cell division in our hypomorphic sgTERT clone, but telomere lengths were nonetheless maintained following cleavage with TRF1-FokI. This indicates that HR can more readily repair telomeres with DSBs within the telomere repeat than the gradually shortened telomere termini that result from the so-called end-replication problem. Importantly, such telomere-internal breaks occur naturally, e.g. during DNA replication of telomeres (Suram and Herbig, 2014), and so it is not surprising that mechanisms for their repair have evolved that can distinguish them from the gradual shortening caused by the end-replication problem. The observation that broken telomeres can be restored by apparent HR mechanisms in cultured human fibroblasts, which lack telomerase activity and undergo senescence due to telomere shortening, is consistent with our findings (Mao et al., 2016).

Murine Zscan4 is a critical regulator of HR-based telomere elongation in mESCs (Zalzman et al., 2010; Lee and Gollahon; 2014, Dan et al., 2017, 2022; Markiewicz-Potoczny et al., 2021). However, we found no evidence that human iPSCs activate a ZSCAN4-dependent transcriptional state in response to targeted telomeric DNA damage. We speculate that this failure to activate ZSCAN4 or other 2C-like genes may reflect a greater similarity of iPSCs to the ‘primed’ mESCs that are derived from postimplantation epiblast, rather than ‘naïve’ mESCs derived from the inner cell mass, and that acquisition of the 2C state may be restricted to naïve pluripotency, though this remains to be tested. Additionally, several key distinctions between our experimental system and others may contribute to this difference: murine vs. human, ESC vs. iPSC, and progressive telomere shortening vs. acute telomere-internal breaks; further studies will be required to better understand the full range of transcriptional responses to telomere dysfunction across different stem cell types.

Very little is known regarding the importance of HR in maintaining iPSC telomeres under normal culture conditions and following damage. It has been shown, e.g. that murine ESC and iPSC telomeres undergo transient ALT-like lengthening during the derivation and reprogramming processes (Liu et al., 2007; Zalzman et al., 2010; Varela et al., 2011; Wang et al., 2012), but it was previously unclear whether HR-based telomere maintenance is employed by human iPSCs and whether fully reprogrammed iPSCs, either murine or human, utilize recombination mechanisms or rely exclusively on telomerase to maintain telomere length following telomeric DNA damage. We found that over several days of TRF1-FokI induction iPSCs undergo negligible telomere shortening, irrespective of telomerase enzymatic activity, and activate telomere recombination in response to targeted telomeric DSBs. Importantly, we observe no telomere length heterogeneity over several days of persistent TRF1-FokI expression, which distinguishes this mechanism of telomere repair from *bona fide* ALT; rather, telomeres appear to be restored to their original length rapidly following cleavage such that any net shortening is undetectable, but they are not lengthened to supraphysiological lengths as they are in ALT+ cell lines. The fact that we observe low levels of ALT-associated PML bodies (APBs) and C-circles in our cells even under uninduced conditions suggests that telomere recombination may play a heretofore underappreciated role in the homeostatic regulation of telomere length in pluripotency, and our finding that many features of ALT significantly increase following acute telomeric DSBs underscores that recombination is an important component of telomere damage repair and length preservation in iPSCs. Furthermore, our findings argue against the concept of a given cell type being labeled strictly ‘ALT+’ or ‘ALT−’ and instead suggest that HR serves as an important telomere damage repair mechanism in cells that are characterized by high telomerase activity, and that these cells may exhibit markers of ALT in the setting of telomere damage without constitutively employing ALT-dependent telomere length maintenance, *per se*.

We propose two potential, and non-exclusive, mechanisms to explain the observed data, both of which would effectively restore broken telomeres to their initial lengths as observed. First, telomere-internal DSBs induced on one sister chromatid behind a replication fork may trigger break-induced replication that uses the telomere on the intact sister chromatid as a template for repair. A weakness of this model is that it is unclear how such breaks would be restricted to this particular phase of the cell cycle. A second potential explanation is that the FokI enzyme induces single-strand nicks within telomeres, which lead to fork collapse and subsequent DSB generation during S phase, followed by efficient replication fork restart. Indeed, TRF1-FokI has been shown to elicit a TIF response selectively during S phase in mouse fibroblasts (Doksani and de Lange, 2016). Although we were unable to obtain direct evidence for the nicking model, it is possible that the steady-state level of such nicks is below our ability to detect them. Consistent with either model, we observed that EME2, which promotes replication fork restart (Pepe and West, 2014), was strongly upregulated in TRF1-FokI-induced iPSCs, as were genes associated with DNA synthesis. We speculate that iPSCs handle FokI-induced fork collapse by undergoing rapid fork restart via robust recombination between sister telomeres, perhaps via a mechanism reminiscent of what is observed in telomerase-negative yeast *nup1* mutants (Aguilera et al., 2020), which enables them to maintain a minimum telomere length sufficient for survival. However, unequal exchanges between sisters underlies the ability of these mutants to maintain telomeres, whereas, we interpret the lack of apparent changes in telomere length to be explained by equal (i.e. in-register) exchanges in human iPSCs.

It is intriguing that iPSCs, which are characterized by high telomerase activity and require it for normal telomere length maintenance, utilize a distinct mechanism to repair telomeric breaks. We speculate that this is a unique characteristic of iPSCs that makes them remarkably adept at both maintaining adequately long and well-protected telomeres while also repairing a type of telomere replication stress that they likely encounter with high frequency, given their highly proliferative nature. Taken together, our study highlights that iPSCs employ specialized responses to telomeric DNA damage and raises important implications for their use as a model to understand normal development, telomere-biology disorders, cancer, tissue regeneration, and cellular aging.

## Materials and methods

### iPSC culture

Wild-type human male iPSC line PENN123i-SV20 was obtained from the University of Pennsylvania Induced Pluripotent Stem Cell Core Facility. Distribution of this cell line was supported by U01TR001810 from the National Institutes of Health (NIH). iPSCs were grown in mTeSR1 complete medium (STEMCELL Technologies) supplemented with 1% penicillin–streptomycin and maintained at 37°C in the presence of 5% O_2_ and 5% CO_2_. iPSCs were cultured on growth factor reduced Matrigel (Corning)-coated plates and passaged in clusters every 4–5 days using StemMACS passaging solution. All cells were routinely screened for mycoplasma contamination using a polymerase chain reaction (PCR)-based assay. For single-cell plating, cells were washed once with Dulbecco’s phosphate-buffered saline (DPBS; Gibco) and incubated with pre-warmed Accutase (STEMCELL Technologies) for 3 min at 37°C, followed by dissociation by pipetting and centrifugation at 200× *g* for 4 min. Cells were then resuspended in mTeSR1 supplemented with 10 μM Thiazovivin (Cayman Chemical) for plating.

### Generation of dox-inducible TRF1-FokI iPSCs

To generate the TRF1-FokI iPSC line, we utilized an established strategy for dox-inducible transgene expression in iPSCs (Sim et al., 2016). The TRF1-FokI sequence was obtained from a lentiviral plasmid gifted by Dr Roger Greenberg. DD-ER-TRF1-FokI (lacking the mCherry sequence present in the original construct) was PCR-amplified and Gibson-cloned into the vector (Addgene, #22074) downstream of the TRE promoter in place of the enhanced green-fluorescence protein (EGFP) reporter. For targeted integration of rtTA and DD-ER-TRF1-FokI into the AAVS1 safe harbor locus, vectors encoding a pair of AAVS1-specific zinc finger nucleases (AAVS1-ZFN-L and AAVS1-ZFN-R) and an AAVS1 Neo-CAG-rtTA donor template were obtained from Addgene (#60915, #60916, and #60431). For transfection, cells were treated with Accutase and seeded at a density of 3.5 × 10^5^ cells per well of a Matrigel-coated 6-well plate to achieve roughly 50% confluency the following day. ZFN-L, ZFN-R, Neo-CAG-rtTA, and sequence-verified Puro-DD-ER-TRE-TRF1-FokI plasmids were transfected into iPSCs using Lipofectamine Stem reagent (Invitrogen) according to the manufacturer's protocol. Transfected cells were allowed to recover for 48 h before being replated as single cells in Matrigel-coated 10-cm plates for clonal selection. Cells underwent dual drug selection with 0.25 μg/ml puromycin for 48 h followed by 40 μg/ml G418 for 10–12 days to select for double targeted clones. Individual clones were expanded, genotyped by PCR using primers targeting both the 5′ and 3′ transgene integration sites, and validated by Sanger sequencing and western blotting.

### TRF1-FokI induction

For all experiments unless otherwise stated, TRF1-FokI iPSCs were pre-treated with 1 μg/ml dox for at least 48 h to allow for maximum expression of TRF1-FokI mRNA. Cells were then induced for the indicated duration with 1 μM Shield-1 ligand (TaKaRa Bio) to stabilize the TRF1-FokI protein and 1 μM 4-OHT (Cayman Chemical) to promote its nuclear internalization. For all time course experiments including RNA-seq, cells were plated at the same density in 6-well plates, pre-treated with dox for 48 h, induced with Shield-1 and 4-OHT in reverse order (i.e. the longest time point induced first), and collected together at the experiment endpoint for assays.

### Generation of TRF1-FokI TP53 KO iPSCs

To generate TRF1-FokI *TP53 KO* iPSCs, the guide RNA (gRNA) sequence 5′-AATGAGGCCTTGGAACTCA-3′ was cloned into the PX330 vector (Addgene, #42230) and transfected into cells using Lipofectamine Stem according to the manufacturer's protocol. Cells were allowed to recover for 48 h after transfection before being replated as single cells in a 10-cm dish for clonal selection with 5 μM Nutlin-3 for 48 h. Individual Nutlin-3-resistant colonies were expanded and p53 knockout was confirmed by western blotting.

### Generation of biallelic hTERT mutant TRF1-FokI iPSCs

To target *hTERT*, four different gRNAs were designed to target exon 10 of the *hTERT* catalytic reverse transcriptase domain, cloned individually into the pCAG-SpCas9-GFP-U6-gRNA plasmid (Addgene, #79144), and transfected into cells using Lipofectamine Stem according to the manufacturer's protocol. Cells were allowed to recover for 40 h after transfection before being FACS-sorted for GFP^+^ cells and replated in 6-well plates. Individual surviving clones were picked, expanded, and screened for loss of telomerase activity by the TRAP assay. More than 50 clones were manually screened, and none showed complete loss of telomerase activity, indicating a strong negative selection against null alleles. The gRNA sequence used to target the sgTERT biallelic mutant clone was 5′-TAGGCTGCTCCTGCGTTTGG-3′, and successful targeting was confirmed by Sanger sequencing.

### Statistical analysis

Unless otherwise noted, all data are representative of at least three independent experiments conducted on different days using cells of different passage derived from the same engineered clone. Statistical analyses were conducted using GraphPad Prism 9.2 software. Quantitative data are expressed as the mean with error bars representing the standard deviation (SD). Student’s *t*-tests (unpaired and two-tailed) or one-way analysis of variance (ANOVA) were used to determine statistical significance for all comparisons where appropriate. Asterisks indicate the degree of significance (*P < 0.05, **P < 0.01, ***P < 0.001, ****P < 0.0001).

## Supplementary Material

mjad058_Supplemental_File

## Notes

**Author contributions:** K.N.E. and F.B.J. conceived the study. K.N.E. conducted all experiments and analyses and wrote and revised the manuscript with editorial suggestions from R.J.F. and F.B.J. J.W.T. conducted bioinformatics analyses related to RNA-seq data. B.M.B. assisted with experiments. F.B.J. supervised the project. All authors read and approved the final manuscript.
