# Supplementary material for: Telomeric DNA breaks in human induced pluripotent stem cells trigger ATR-mediated arrest and telomerase-independent telomere damage repair
Source: J Mol Cell Biol. 2023 Sep 28;16(3):mjad058. doi: 10.1093/jmcb/mjad058 (PMC11429528; doi:10.1093/jmcb/mjad058)
Supplement: mjad058_Supplemental_File [file mjad058_supplemental_file.pdf]

SUPPLEMENTARY INFORMATION

Telomeric DNA breaks in human induced pluripotent stem cells trigger ATR-mediated arrest and telomerase-independent telomere damage repair

Katrina N Estep, John W Tobias, Rafael J Fernandez III, Brinley M Beveridge, F Brad Johnson

SUPPLEMENTARY Materials and methods

EdU staining using Click-iT™ EdU Imaging Kit

EdU staining was performed using the Invitrogen Click-iT™ EdU Imaging Kit according to the manufacturer's instructions. Briefly, TRF1-FokI iPSCs were seeded on Matrigel-coated chamber slides in 1 μg/ml dox 48 hours prior to the start of the experiment. Cells were then induced with 1 μM Shield-1 and 1 μM 4-OHT for increasing amounts of time (up to 72 hours). EdU was added at a final concentration of 10 μM for 30 minutes before slides were fixed in 4% paraformaldehyde (PFA), washed, permeabilized, and incubated with 1× Click-iT reaction cocktail containing Alexa Fluor 488 for 30 minutes at room temperature protected from light. Following incubation, slides were washed, counterstained with DAPI, air dried, mounted using Prolong Gold Antifade overnight, and imaged on a Leica DM6000 widefield fluorescent scope.

Propidium iodide and intracellular phospho-H3 flow cytometry

Cells were washed once with 1× DPBS, treated with pre-warmed Accutase to generate single cells, and collected and centrifuged in mTeSR1. Pelleted cells were resuspended in 500 μl of 1× DPBS and fixed in 5 ml ice cold 70% EtOH added slowly while vortexing to minimize cell clumping. Cells were allowed to fix overnight at 4°C. For propidium iodide (PI) staining, cells were pelleted, washed in 1× DPBS, and resuspended in 200 μl of permeabilization solution (0.1% Triton-X in DBPS) containing 10 μl of eBioscience™ propidium iodide (Invitrogen) and 0.5 μg RNase A. Staining and RNA digestion was carried out for 1 hour at room temperature protected from light. Cells were filtered and analyzed on an Accuri C6 analyzer. For phospho-H3 staining, cells were permeabilized after fixation in 0.1% Triton-X in DPBS for 10 minutes and blocked in 1× DPBS containing 3% BSA and 0.1% Tween-20 for 30 minutes at room temperature. Cells were then incubated with 200 μl of 1× DPBS + 1% BSA + 0.1% Tween-20 containing 4 μl of Alexa Fluor 488-conjugated phospho-histone H3 (Ser10) antibody (Cell Signaling Technology, 1:50 dilution) for 90 minutes at room temperature, washed, filtered, and analyzed on an Accuri C6 analyzer. All data were quantified using FlowJo software, with manual gating for cell cycle distribution.

Annexin V and SA β-galactosidase flow cytometry

Cells were washed once with 1× DPBS, treated with pre-warmed Accutase to generate single cells, and collected and centrifuged in mTeSR1. For Annexin V staining, pelleted cells were washed in cold PBS and then resuspended in 1× binding buffer (10 mM HEPES pH 7.4, 140 mM NaCl, 2.5 mM $CaCl_2$) at a density of 1 x 10^6 cells/ml. 100 μl of this suspension (1 x 10^5) was transferred to a 5 ml culture tube and 5 μl of FITC Annexin V (BD Biosciences) added. Cells were stained at room temperature for 30 minutes protected from light and analyzed on an Accuri C6 analyzer using the FL-1 filter setting within 30 minutes of cell collection. SA β-galactosidase staining was performed using the CellEvent™ Senescence Green Flow Cytometry Assay Kit (Fisher). Single cells were collected, spun down, washed in 1× DPBS, fixed in 2% PFA for 10 minutes, washed, and resuspended in working solution containing 1× Senescence Green Probe diluted in CellEvent™

binding buffer. Cells were incubated for 1 hour at 37°C in the absence of CO_2 and protected from light. After incubation the working solution was removed and cells were washed with 1% BSA in PBS and analyzed on a BD Accuri C6 analyzer using a 488 nm laser and FL-1 filter setting. All data were quantified using FlowJo software.

Immunofluorescence staining and microscopy

Cells were seeded on Matrigel-coated chamber slides (Nest Scientific) for immunofluorescence staining. Slides were washed once with 1× DBPS, fixed in 4% PFA for 10 minutes at room temperature, washed, permeabilized in 0.5% Triton-X in DPBS for 5 minutes at room temperature, blocked in 3% BSA in 0.1% Triton-X in DPBS for 30 minutes at room temperature, and incubated overnight at 4°C in primary antibody diluted in 1% BSA and 0.1% Tween-20 (for list of antibodies, see Key Resources table). Following primary antibody incubation, slides were washed 3 times in 1× DPBS and incubated with a fluorophore-conjugated secondary antibody for 1 hour at 37°C. Secondary antibody-only (no primary antibody) slides were included as negative staining controls. Following secondary antibody incubation, slides were washed 3 times in PBST (0.1% Tween-20) with 1 μg/ml DAPI added to the penultimate wash. Slides were air dried and mounted in Prolong Gold antifade reagent (Invitrogen) overnight. Images were acquired using either a Nikon Eclipse E600 or a Leica DM6000 widefield fluorescent microscope. Images were prepared and analyzed using Fiji software. Antibodies are listed in Table S1.

Western blotting

Lysates were prepared by resuspending fresh or previously frozen cell pellets in RIPA lysis buffer (50 mM Tris-HCl pH 7.4, 150 mM NaCl, 1% Triton-X, 1% sodium deoxycholate, 0.1%-0.2% SDS) supplemented with 1× protease inhibitor cocktail (Roche) and 1× phosphatase inhibitor (Cell Signaling Technology). Cells were lysed on ice for 30 minutes with intermittent vortexing, then centrifuged at 12,000 rpm for 5 minutes at 4°C to pellet cell debris. Supernatant was collected and protein concentration was quantified by Bradford assay using protein assay dye (Bio-Rad) absorbance at 595 nm. 20 μg of protein per sample was resolved on a NuPage 4-12% Bis-Tris polyacrylamide mini gel (Bio-Rad) and transferred to a nitrocellulose membrane in Towbin transfer buffer containing 20% MeOH at 100V for 1 hour or 25V for 16 hours. Membranes were blocked in TBST (1× TBS with 0.1% Tween-20) containing 5% nonfat milk, incubated with primary antibody diluted in blocking solution overnight at 4°C with nutation, rinsed 3 times for 5-10 minutes in TBST, incubated in secondary antibody diluted in blocking solution for 1 hour at room temperature with nutation, and rinsed 3 times for 5-10 minutes in TBST. Membranes were then exposed to Pierce ECL Western Blotting Substrate (Thermo) and imaged on a Typhoon-FLA scanner. Antibodies are listed in Table S1.

Cell viability by Cell Titer Blue™ assay

Cell Titer Blue™ reagent was used to measure relative cell viability. Briefly, cells were seeded at equivalent densities in mTeSR1 supplemented with 10 μM Thiazovivin and 1 μg/ml dox in multiple wells of a Matrigel-

coated 24-well plate and allowed to recover for 48 hours before Thiazovivin was withdrawn. Negative control wells were included which contained Matrigel and medium but no cells. Wells were then drugged in triplicate for up to 72 hours with 1 μg/ml dox, 1 μM Shield-1, and 1 μM 4-OHT to induce expression of TRF1-FokI and any other small molecules were added to the assay at the time of induction. At the end of the induction period Cell Titer Blue reagent was added at a final dilution of 1:5 (125 μl per 500 μl of medium in each well), mixed gently, and incubated at 37°C for 2 hours. Fluorescence was recorded at 560/590 nm using a Tecan Infinite 200 Pro plate reader and the average background fluorescence subtracted from each test sample.

Telomere length measurement by terminal restriction fragment (TRF) analysis

DNA was isolated from cells using a Gentra Puregene core kit A (Qiagen) and quantified by both Nanodrop and quantitative fluorometry using QuBit 2.0 (Invitrogen). For TRF analysis, 500-1000 ng of DNA was restriction digested with CviAII overnight at 25°C followed by digestion with a mixture of BfaI, MseI, and NdeI for 8 hours at 37°C. For samples that were additionally treated with S1 nuclease (Invitrogen), 500 ng of restriction digested DNA was treated in 30 mM sodium acetate pH 4.6, 1 mM zinc acetate, 5% glycerol for 30 minutes at 37°C. Southern blotting was carried out using previously established protocols with some modification (Kimura et al., 2010). Conventional TRF analyses were resolved on a 0.7% agarose gel at 0.833 V/cm for 16-24 hours. Pulsed field gel electrophoresis was performed in a 1.2% agarose gel (pulse field certified agarose, Bio-Rad) in 0.7× TAE at 5.8 V/cm and with a 1-to-8 second switch time over 16 hours in a CHEF-DRII apparatus (Bio-Rad). For Southern blot analysis, gels were depurinated and denatured and then transferred to a Hybond XL membrane (Cytiva) overnight by capillary transfer using denaturation buffer. The Hybond membrane was hybridized using a DIG-labeled telomere probe at 42°C overnight. The blot was then washed and exposed using CDP-Star on a LAS-4000 Image Quant imager. TRFs were analyzed using ImageQuant.

Immunofluorescent staining of metaphase chromosomes (meta-TIF)

Cells were treated with 0.2 μg/ml colcemid for 90 minutes in to arrest cells in metaphase. Colcemid medium was collected and cells were washed once, then dissociated in pre-warmed Accutase, collected into the same conical and pelleted. Supernatant was removed and cells were gently resuspended by flicking in 0.5 ml of residual medium. Cells were swelled in 10 ml of hypotonic solution (0.2% KCl, 0.2% trisodium citrate) at room temperature for 10 minutes and then spun onto poly-L-lysine coated glass slides at 2000 rpm for 10 minutes using a Shandon Cytospin 3. Slides were then fixed in 4% PFA in PBS for 10 minutes at room temperature, washed in 1× DPBS, permeabilized for 10 minutes at room temperature in KCM buffer (120 mM KCl, 20 mM NaCl, 10 mM Tris-HCl pH 7.5) containing 0.1% Triton-X, and blocked with blocking buffer containing 2% BSA and 100 µg/ml RNase A at 37°C for 15 minutes. Slides were incubated with primary antibody for 1 hour at 37°C, washed 3 times in 1× PBST, incubated with secondary antibody for 1 hour at room temperature, and washed 3 times again in 1× PBST with DAPI added to the penultimate wash. Slides were then air dried,

mounted in Prolong Gold overnight, and imaged using a Leica DM6000 widefield fluorescent microscope. Images were prepared and analyzed using Fiji software.

Telomere CO-FISH

For telomere CO-FISH, iPSCs were incubated with 7.5 μM BrdU and 2.5 μM BrdC for 8 hours prior to induction of TRF1-FokI. TRF1-FokI was then induced for 4 hours or mock induced by addition of DMSO. 0.2 μg/ml colcemid was added for the last 2 hours in to arrest cells in metaphase. Medium was collected and cells were washed once, then dissociated in pre-warmed Accutase, collected into the original medium and centrifuged to pellet. Supernatant was removed and cells were gently resuspended by flicking in 0.5 ml of residual medium. Cells were swelled in 10 ml of prewarmed 0.075 M KCl hypotonic solution at 37°C for 15 minutes and then prefixed by addition of 100 μl MeOH/acetic acid (3:1) fixative solution and pelleted. Metaphase cells were then fixed by washing 3 times in 10 ml of cold MeOH/acetic acid (3:1) and stored at 4°C until ready to spread. Spreading was accomplished by manually dropping fixed metaphase cells onto poly-L-lysine-coated glass slides tilted at a 45-degree angle over a steaming beaker of water and blowing on the slides to spread the cells. Slides were air dried overnight at room temperature. On the day of CO-FISH staining, slides were rehydrated in 1× DPBS for 5 minutes, then treated with 0.5 mg/ml RNase A in PBS for 15 minutes at 37°C, rinsed in 1× DPBS, stained with 0.5 μg/ml Hoechst in 2× SSC buffer for 20 minutes at room temperature, and rinsed once more. Slides were then placed in a shallow plastic tray, covered with a thin layer of 2× SSC, and exposed to 365-nm UV light using a UV Stratalinker 1800 (Stratagene) set to 5400 μJ x100 at room temperature. Following UV exposure, BrdU/BrdC-labeled DNA was digested with 10 U/μl Exonuclease III (Promega) in buffer supplied by the manufacturer for 30 minutes at 37°C. Slides were then washed once in 1× DPBS for 5 minutes, fixed in 4% PFA, rinsed in 1× DPBS again, dehydrated in a 70%, 85%, 100% EtOH series for 2 minutes each, and air dried. Hybridization of the first PNA probe (TelC-Cy3) was then carried out in hybridization solution (70% deionized formamide, 0.25% blocking reagent (Roche), 10 mM Tris-HCl pH 7.2) for 2 hours at room temperature. Following the first hybridization, slides were washed twice in PNA wash buffer (70% deionized formamide, 10 mM Tris-HCl pH 7.2, 0.1% BSA). The second hybridization step was carried out by incubating slides with the second PNA probe (TelG-488) diluted in hybridization solution for another 2 hours at room temperature. Lastly, hybridized slides were washed twice with PNA wash buffer for 15 minutes at room temperature on a shaker, washed 3 times with wash buffer II (0.1M Tris-HCl pH 7.2, 0.15M NaCl, 0.08% Tween-20) in a Coplin jar for 5 minutes at room temperature with 1 μg/ml DAPI added to the penultimate wash, air dried, and mounted in Prolong Gold overnight. Stained metaphases were imaged using a Leica DM6000 widefield fluorescent microscope. Images were prepared and analyzed using Fiji software.

RNA sequencing

For RNA sequencing, TRF1-FokI iPSCs and parental unedited iPSCs were seeded in triplicate at a density of 25,000 single cells per well of triplicate Matrigel-coated 6-well plates in 1 μg/ml dox and 2 μM Thiazovivin.

Cells were allowed to recover for 48 hours prior to induction, and Thiazovivin was withdrawn on the morning of the second day. TRF1-FokI expression was then induced by addition of 1 μM Shield-1 and 1 μM 4-OHT for 8, 24, or 48 hours, then cells were collected by lysing directly in the plate with TriZol reagent (Fisher) at the experiment endpoint and flash frozen in liquid nitrogen. Total RNA was precipitated from TriZol (Ambion) and cleaned using the Monarch RNA Cleanup Kit (NEB). RNA integrity was verified by the Penn Genomic Analysis Core and only RNAs with RIN>9 were used. RNA library preparation and sequencing was performed by the Penn Next Generation Sequencing Core Facility according to standard protocols. Briefly, high-throughput library prep was performed using Illumina TruSeq stranded mRNA kit and library quality checked on an Agilent Bioanalyzer. Sequencing was performed on a NovaSeq whole S1 flowcell using a 100-cycle kit. For analysis, Salmon was used to count the data against the transcriptome defined in Gencode v41. On a local workstation, several Bioconductor packages in R were used for subsequent steps. The transcriptome count data was annotated and summarized to the gene level with tximeta and further annotated with biomaRt. PCA analysis and plots were generated with PCAtools. Normalizations and statistical analyses were done with DESeq2. GSEA pathway analysis was done against the indicated gene sets from the Molecular Signatures Database (MSigDB), using the DESeq2 statistic as a ranking metric. All GSEA analyses were run with either 10,000 or 20,000 permutations and a weighted enrichment statistic.

Telomerase activity measurement by the TRAP assay

Relative telomerase activity was measured by Telomere Repeat Amplification Protocol (TRAP) assay as previously described (Herbert et al., 2006). In brief, cells were harvested by Accutase, pelleted, and lysed in 1× CHAPS buffer for 30 minutes on ice. Lysates were then centrifuged at 16,000g for 20 minutes to pellet cell debris and protein concentration was measured by Bradford assay. Serial dilutions of lysate were incubated with a telomerase substrate at 30°C for 30 minutes to allow for telomerase to catalyze the addition of telomere repeats to the substrate. The reactions were then PCR amplified, resolved on a 4%-20% TBE polyacrylamide gel and visualized by staining with SYBR Green nucleic acid gel stain. Relative telomerase activity was quantified using ImageJ software.

C-circle assay

Genomic DNA was isolated by phenol–chloroform precipitation and quantitated using a Qubit 2.0 fluorometer. C-circle reactions were carried out as previously described (Henson et al., 2017). Briefly, 40 ng DNA was reconstituted in 10 μl of TE in a PCR tube, then 9.25 μl of a mastermix containing 2.16× Phi29 DNA polymerase buffer, 0.216% Tween-20, 8.65 μg/ml recombinant albumin, and 2 mM each of dATP, dGTP, dTTP, and dCTP was aliquoted to each tube and mixed. U2OS DNA was included as a positive control and HEK293T DNA was included as a negative control. Each assay also included no DNA controls and a set of reactions lacking Phi29 polymerase to account for background. To remove linear genomic DNA, DNA samples were digested with a mixture of *Cvi*AII, *Bfa*I, *Mse*I, and *Nde*I for 16 hours at 25°C followed by 8 hours at 37°C, then digested with 12.5 U Lambda Exonuclease (NEB) and 100 U Exonuclease I (NEB) for 2 hours at 37°C.

Exo-digested DNAs were ethanol precipitated, resuspended in TE, and analyzed on a Southern blot for telomere repeats to confirm complete digestion of linear telomeric DNA. Rolling circle amplification for C-circle detection was carried out at 30°C for 8 hours, followed by a 20-minute incubation at 70°C to inactivate the polymerase. Reactions were then dot blotted onto a Hybond-XL membrane under native conditions, UV-crosslinked, and hybridized using a DIG end-labeled C-rich telomere probe at 42°C overnight. The blot was then blocked, incubated with anti-DIG antibody, washed, and exposed using CDP-Star on a LAS-4000 Image Quant imager.

SUPPLEMENTARY References

Henson, J. D., Lau, L. M., Koch, S., Martin La Rotta, N., Dagg, R. A., & Reddel, R. R. (2017). The C-circle assay for alternative-lengthening-of-telomeres activity. *Methods (San Diego, Calif.)*, *114*, 74–84. https://doi.org/10.1016/j.ymeth.2016.08.016

Herbert, B.-S., Hochreiter, A. E., Wright, W. E., & Shay, J. W. (2006). Nonradioactive detection of telomerase activity using the telomeric repeat amplification protocol. In *Nature Protocols* (Vol. 1, Issue 3, pp. 1583–1590). https://doi.org/10.1038/nprot.2006.239

Kimura, M., Stone, R. C., Hunt, S. C., Skurnick, J., Lu, X., Cao, X., Harley, C. B., & Aviv, A. (2010). Measurement of telomere length by the Southern blot analysis of terminal restriction fragment lengths. *Nature Protocols*, *5*(9), 1596–1607. https://doi.org/10.1038/nprot.2010.124

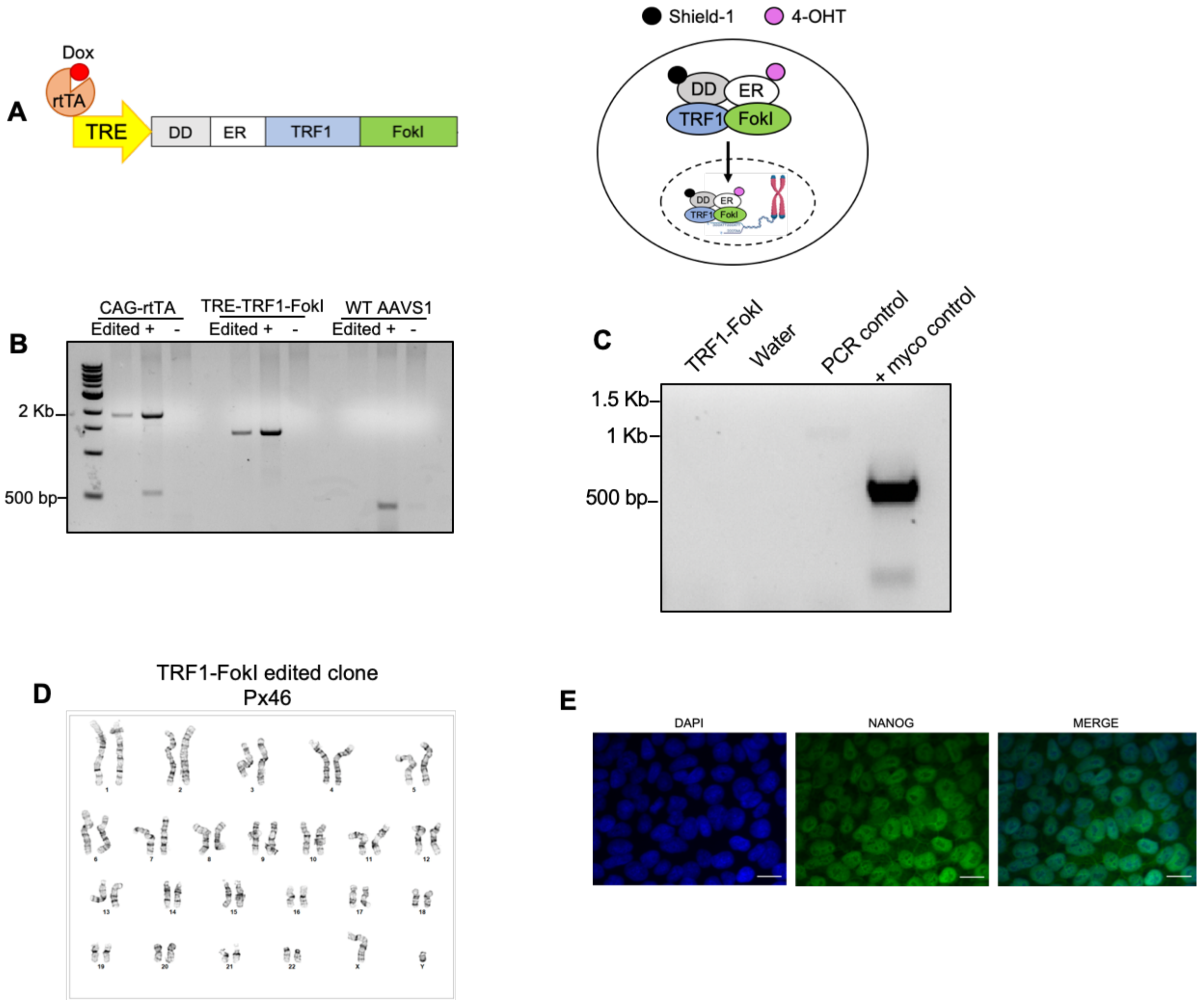

Figure S1: Validation of engineered TRF1-FokI iPSC line

A) TRF1-FokI construct design. Upon addition of doxycycline, the constitutively expressed rtTA binds dox and drives transcription of the TRE-regulated DD-ER-TRF1-FokI allele. Once the protein is translated, Shield-1 ligand binds the DD domain to stabilize the cytosolic pool of protein and 4-OHT binds the ER domain to allow the stabilized protein to enter into the nucleus, where the TRF1 domain localizes the protein to telomere repeats to introduce telomeric double strand breaks.

B) Genotyping PCR confirming the integration of CAG-rtTA and TRE-DD-ER-TRF1-FokI repair templates into the AAVS1 locus.

C) PCR-based test to confirm the edited TRF1-FokI clone is negative for mycoplasma.

D) The edited TRF1-FokI iPSC clone shows normal karyotype following the integration of rtTA and TRF1-FokI constructs into AAVS1.

E) The edited TRF1-FokI iPSC clone maintains expression of Nanog, a marker of pluripotency. (Scale bar=10 μm).

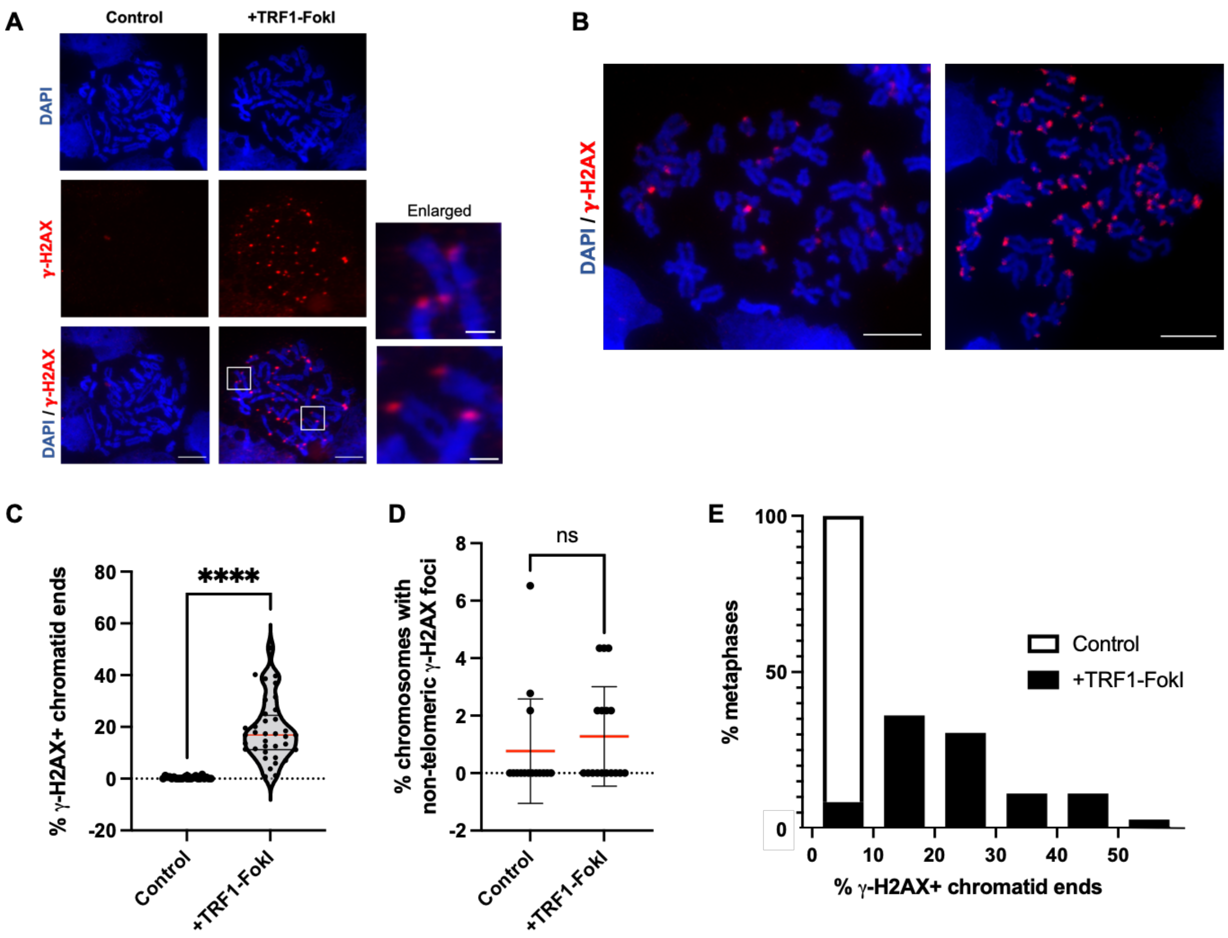

Figure S2: TRF1-FokI DSBs are restricted to telomeres

A) Immunofluorescent staining for **γ**-H2AX in uninduced cells and cells induced to express nuclear TRF1-FokI for 4 hours. Experimental setup is identical to that shown in Figure 1G. Left two panels: scale bar = 10 μM. Enlarged panels: scale bar = 2 μM.

B) Representative examples of metaphase spreads with varying frequencies of telomeric **γ**-H2AX foci. Scale bar = 10 μM.

C) Quantification of meta-TIF staining from 3 independent experiments. At least 30 spreads were analyzed per condition for a total of >1000 chromosomes. P-value is from two tailed unpaired t-test ($p<0.0001$).

D) Quantification of non-telomeric **γ**-H2AX foci in metaphase chromosomes stained as in C. Data represent the mean and SD from 3 independent experiments. P-value is from two tailed unpaired t-test ($p=0.419$).

E) Frequency distribution showing the percentage of metaphases analyzed that exhibited the indicated bin percentage of **γ**-H2AX positive chromatid ends (0-10%, 10-20% etc.). For example, 100% of control metaphases exhibited <10% **γ**-H2AX positive chromatid ends.

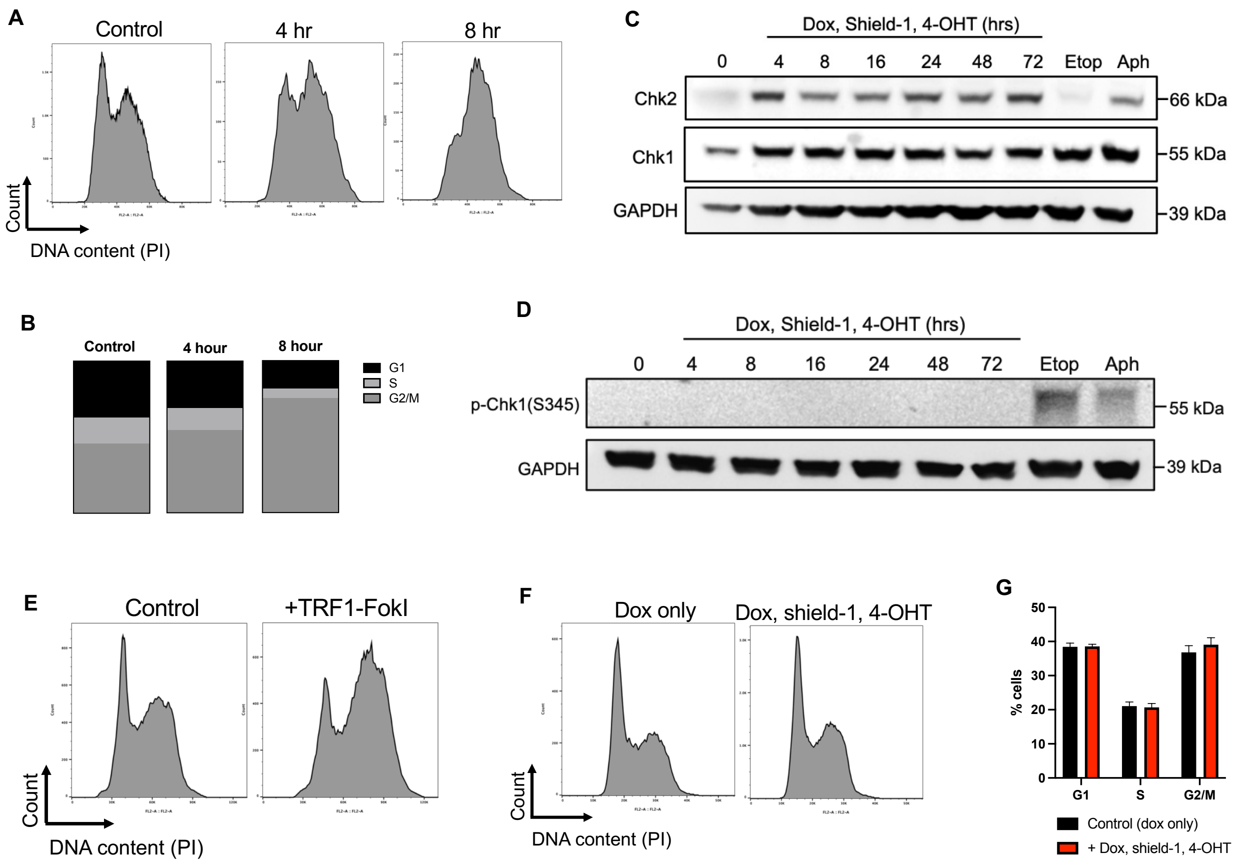

Figure S3: DNA damage signaling and cell cycle analysis in TRF1-FokI induced iPSCs

A) Quantification of cell cycle analysis of TRF1-FokI iPSCs uninduced (dox only) and induced (dox, Shield-1, 4-OHT) for 4 and 8 hours as measured by propidium iodide flow cytometry. Data are representative of averages from 3 independent experiments.

B) Representative histogram of DNA content of TRF1-FokI iPSCs uninduced (dox only) and induced (dox, Shield-1, 4-OHT) for 4 and 8 hours as measured by propidium iodide flow cytometry.

C) Western blot analysis of total CHK1 and CHK2 protein levels following induction of TRF1-FokI for the indicated amount of time or after treatment with high dose etoposide (10 μM) or aphidicolin (10 μM) for 2 hours. Lysates are the same as those in Fig. 2A.

D) Western blot analysis of phospho-Chk1(S345) levels following induction of TRF1-FokI for the indicated amount of time or after treatment with high dose etoposide (10 μM) or aphidicolin (10 μM) for 2 hours. Lysates are the same as those in Fig. 2B.

E) Representative cell cycle profile measuring the effect of Chk1i inhibition on TRF1-FokI uninduced (dox only, left) and induced (dox, Shield-1, 4-OHT) iPSCs. Cells were co-treated at the time of induction with 10 nM Chk1 inhibitor Prexasertib.

F) Representative cell cycle profile of unedited parental SV20 iPSCs treated with dox only or complete induction medium containing dox, Shield-1, and 4-OHT as measured by propidium iodide flow cytometry.

G) Quantification of flow cytometry data from C. Data are representative of average and SD from 2 independent experiments.

A

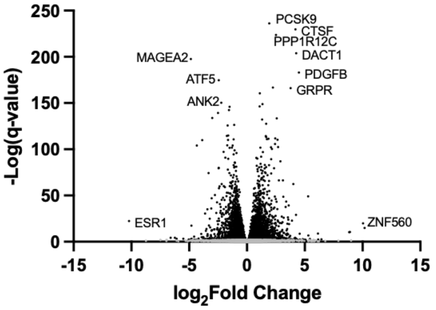

B

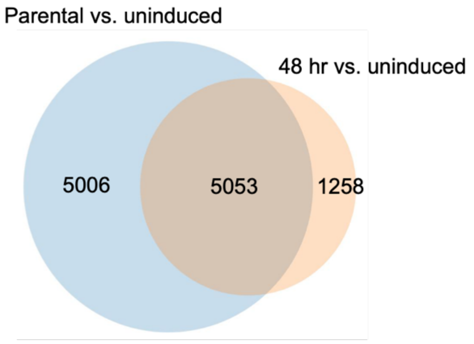

C

Genes upregulated			Genes downregulated		
Gene_name	log2FoldChange	padj	Gene_name	log2FoldChange	padj
PCSK9	1.926104305	7.111E-237	MAGEA2	-4.860260016	2.843E-198
CTSF	4.195884043	1.941E-230	ATF5	-2.441814385	1.81E-175
PPP1R12C	2.512843901	1.792E-224	ANK2	-2.225923793	3.942E-151
DACT1	4.263586369	1.163E-204	HSPA1A	-1.513927135	9.44E-147
PDGFB	4.493405431	8.217E-184	SFPQ	-1.553237308	3.996E-143
CCN2	2.233518126	1.362E-167	NCOA5	-2.502198496	6.513E-140
GRPR	3.770144836	7.907E-167	MAP1A	-3.014631265	1.386E-134
JADE1	1.10804512	3.107E-161	UBTF	-1.108810136	5.396E-127
PDLIM1	1.34083888	1.505E-149	ODC1	-1.681283275	1.619E-126
HK1	1.097413117	3.895E-147	BEND3	-1.621610617	1.106E-115
STARD8	2.57572245	3.543E-134	HSPA1B	-1.738046018	2.768E-115
PYGL	1.30192692	6.886E-133	HEXIM1	-1.15003973	2.102E-111
CORO2A	1.658402829	2.389E-128	PSAT1	-1.648286471	8.476E-111
SH2D3A	1.62829841	4.779E-115	CUZD1	-3.866791799	1.225E-110
HHLA1	1.663497688	4.748E-114	UHRF1	-1.706701413	5.801E-105
BTG2	2.436143063	1.79E-112	CSAG3	-4.349278805	8.889E-105
RAB20	1.86047669	3.671E-112	RBM12	-1.241075799	1.06E-104
KCNK6	1.730349592	1.234E-111	MAT2A	-1.310817804	1.433E-102
NEDD9	2.63447555	5.818E-111	ZBTB12	-1.510975848	3.366E-102
SLC16A3	2.42979308	1.901E-106	DYNLL1	-1.066730804	1.724E-99

D

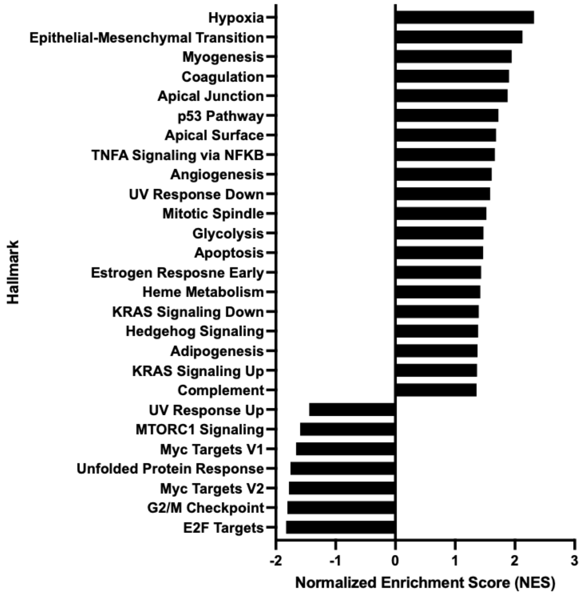

Figure S4: Pathways differentially expressed in parental iPSCs cultured under induction conditions vs. uninduced TRF1-FokI iPSCs

A) Volcano plot showing genes up- or down-regulated in unedited parental cells treated for 48 hours with dox, Shield-1, and 4-OHT compared to uninduced TRF1-FokI cells treated with dox only.

B) Venn diagram indicating the number of significantly (<0.05) differentially expressed lncRNA-, miRNA-, and protein-coding genes in each control condition. Based on these findings, drug-treated parental cells were selected as the control group for RNA-seq analyses.

C) Lists of the top 20 upregulated (left) and downregulated (right) genes in parental cells vs. uninduced TRF1-FokI iPSCs.

D) Gene set enrichment analysis (GSEA) of pathways significantly (<0.05) up- and down-regulated in parental vs. uninduced TRF1-FokI iPSCs.

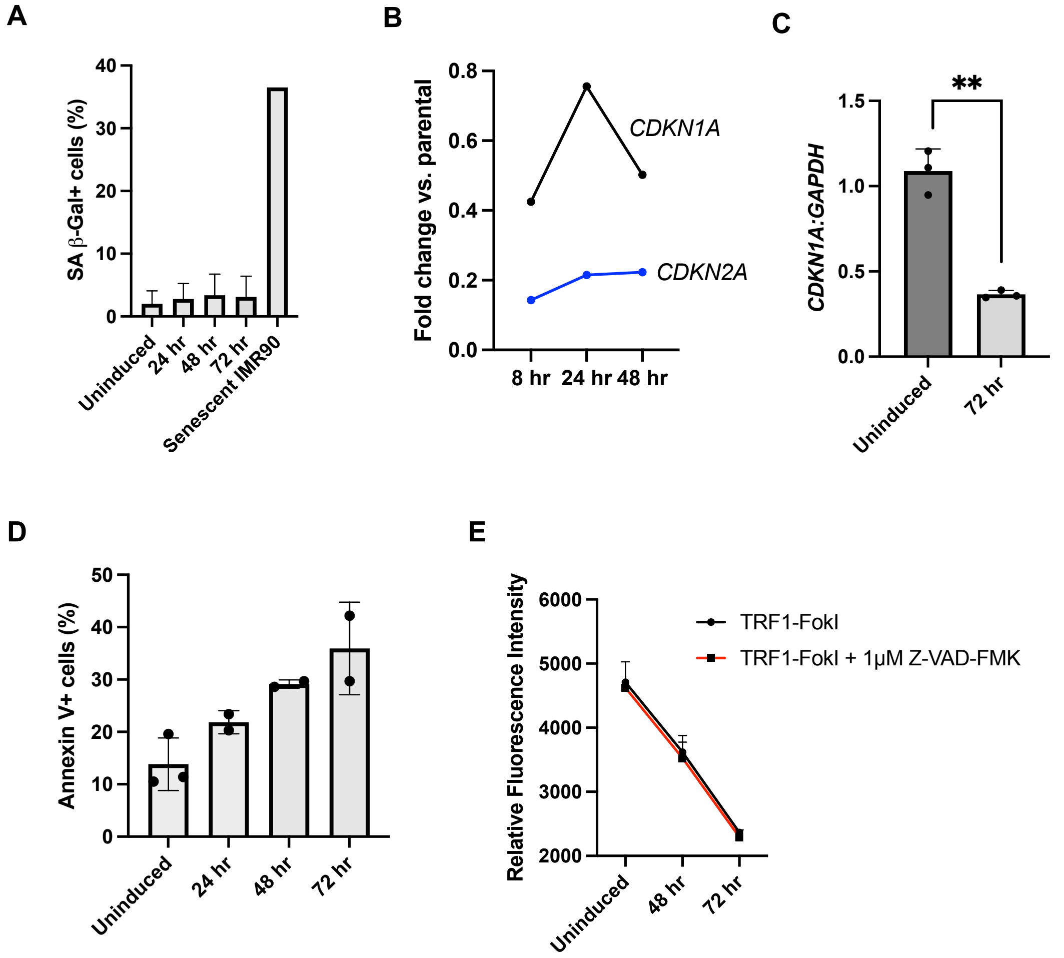

Figure S5: Analysis of the effects of long-term induction of TRF1-FokI in iPSCs

A) SA B-gal+ cells measured by flow cytometry across 72 hours of induction. IMR90 fibroblasts passaged to replicative senescence (px78) serve as a positive control (N=1). Data represent the mean and SD from 2 independent experiments.

B) Relative expression levels of senescence regulators p21 (CDKN1A) and p16 (*CDKN2A*) across 48-hour induction time course as measured by RNA seq compared to unedited parental cells.

C) Quantification of expression of *CDKN1A* mRNA transcripts at 72 hours of induction as measured by RT-qPCR and normalized to *GAPDH*. Data represent the mean and SD calculated from 3 independent experiments from 3 biological replicate samples, each containing triplicate qPCR reactions per time point.

D) Annexin V+ cells measured by flow cytometry across 48 hours of induction. Data represent the mean and SD from 2 independent experiments.

E) Fluorometric cell viability assay of TRF1-FokI cells across 72 hours of induction with additional treatment with 1 μM pan-caspase inhibitor Z-VAD-FMK at the time of induction. Data represent the mean and SD from triplicate samples per condition measured at each time point.

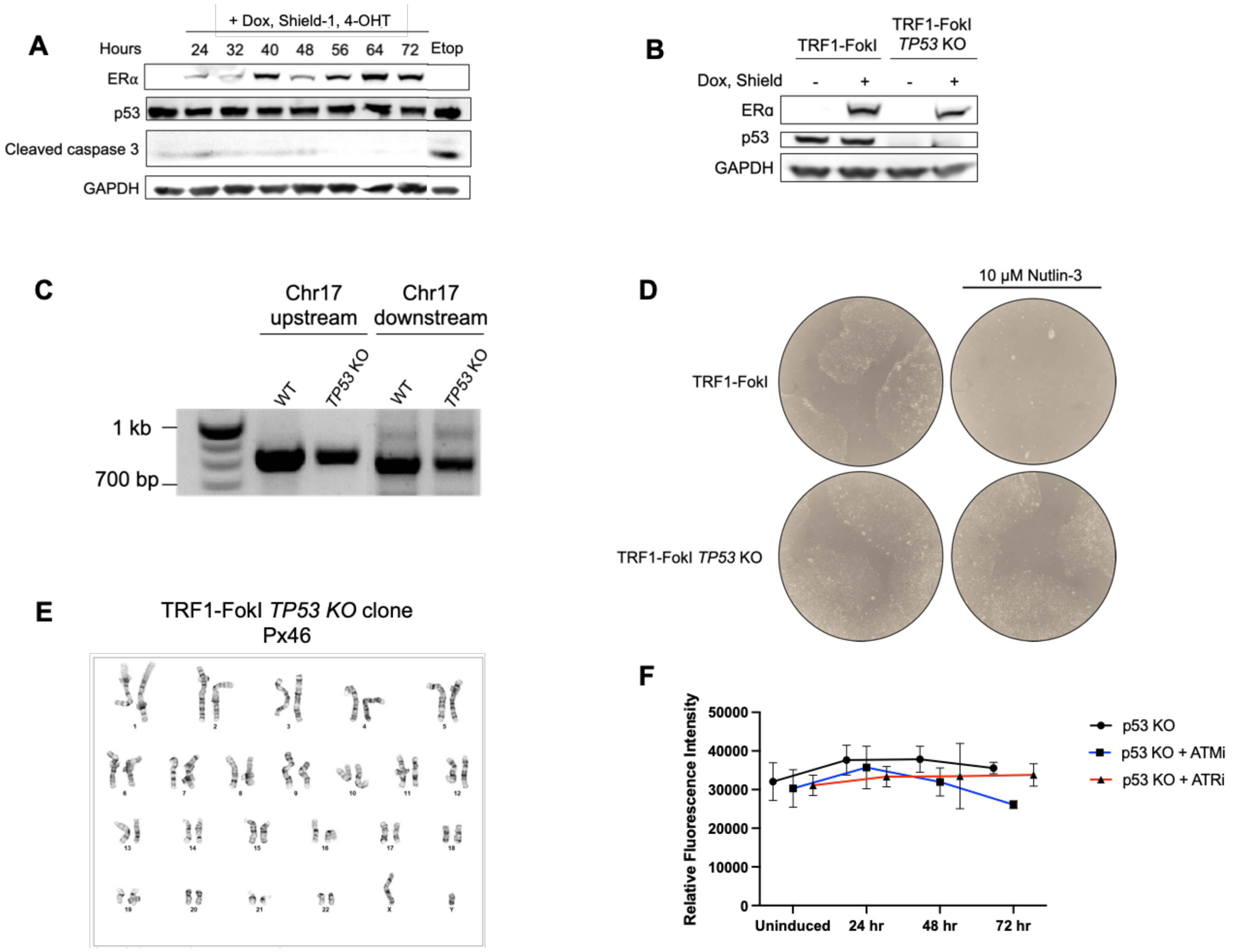

Figure S6: Generation of TRF1-FokI *TP53* KO cells

- **A)** Western blot analysis of total p53 protein levels in WT TRF1-FokI iPSCs across 96 hours of TRF1-FokI induction and in the same cells treated with 10 μM etoposide for 2 hours.
- **B)** Western blot confirmation of p53 knockout in TRF1-FokI iPSCs.
- **C)** Genotyping PCR of parental *TP53* WT TRF1-FokI line and *TP53 KO* clone using two primer sets designed against chromosome 17 upstream and downstream of the gRNA site in exon 10 of the *TP53* gene. Presence of a band in both PCRs confirms CRISPR deletion is contained within the TP53 coding sequence. For primer sequences see key resources table.
- **D)** Nutlin-3 viability assay to screen for loss of p53 function in expanded TRF1-FokI *TP53* KO cell line.
- **E)** Karyotype analysis of TRF1-FokI *TP53* KO edited clone. The cell line exhibits normal karyotype.
- **F)** Fluorometric cell viability assay of TRF1-FokI *TP53 KO* cells across 72 hours of induction with additional treatment with ATMi or ATRi. Data represent the mean and SD from triplicate samples per condition measured at each time point.

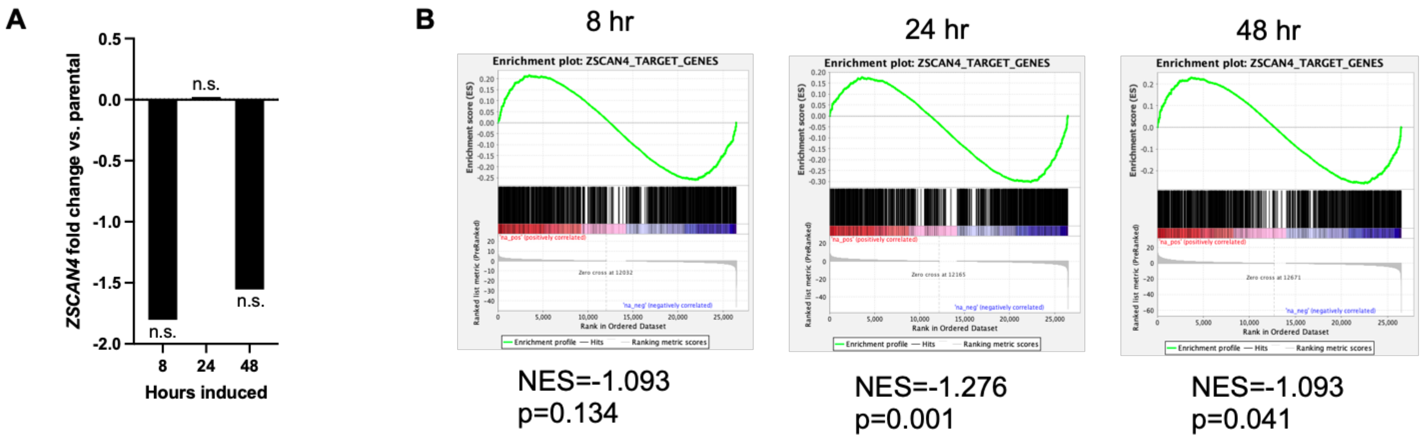

C

Symbol (mouse)	Ortholog (human)	log2FoldChange (48 hr vs. parental)	p-value
Gm16429	N/A		
Inpp4b	INPP4B	-0.446883731	0.63401593
Tdpoz4	SPOP	0.46467599	0.00183055
Tcstv3	N/A		
Gm10696	N/A		
Zfp352	KLF18	-0.969487721	0.42094566
Zscan4b			
Zscan4d	ZSCAN4	-1.555680831	0.55764993
Gm6351			
Gm12794	PRAMEF1	Not detected	
AF067061	N/A		
Gm4303			
Gm4302	ALYREF	0.414618569	0.00036532
Spesp1	SPESP1	-1.326978957	0.77056715

Figure S7: Human orthologues of mESC 2 cell-like genes are not enriched in TRF1-FokI induced iPSCs

A) Relative *ZSCAN4* mRNA levels normalized to parental throughout 48 hours of TRF1-FokI induction.

B) GSEA indicating that the ZSCAN4 target gene set is significantly downregulated at 48 hours of TRF1-FokI induction.

C) Table indicating murine 2C-genes enriched in mESCs following deletion of TRF2 [13], with human orthologues and their relative expression as detected by RNA-seq at 48 hours of TRF1-FokI induction compared to parental unedited cells.

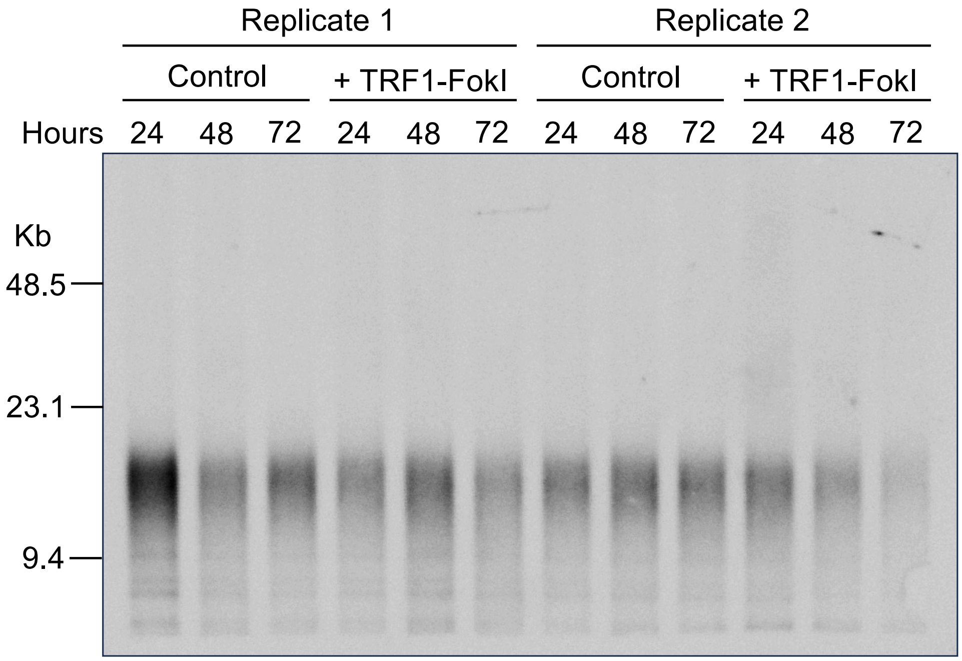

Figure S8: TRF1-FokI-induced cells do not exhibit detectable telomere shortening

TRF blot of genomic DNA resolved by pulse-field gel electrophoresis. DNA samples from two independent sets of cultures either lacking (control, dox-treated only) or expressing the TRF1-Fok1 transgene (+TRF1-FokI) were analyzed, and TRF-FokI was induced in the same fashion as in Figure 5A for the times indicated. Note that although there is some variability in the amount of DNA loaded among the lanes, there is no apparent shortening caused by TRF1-Fok1.

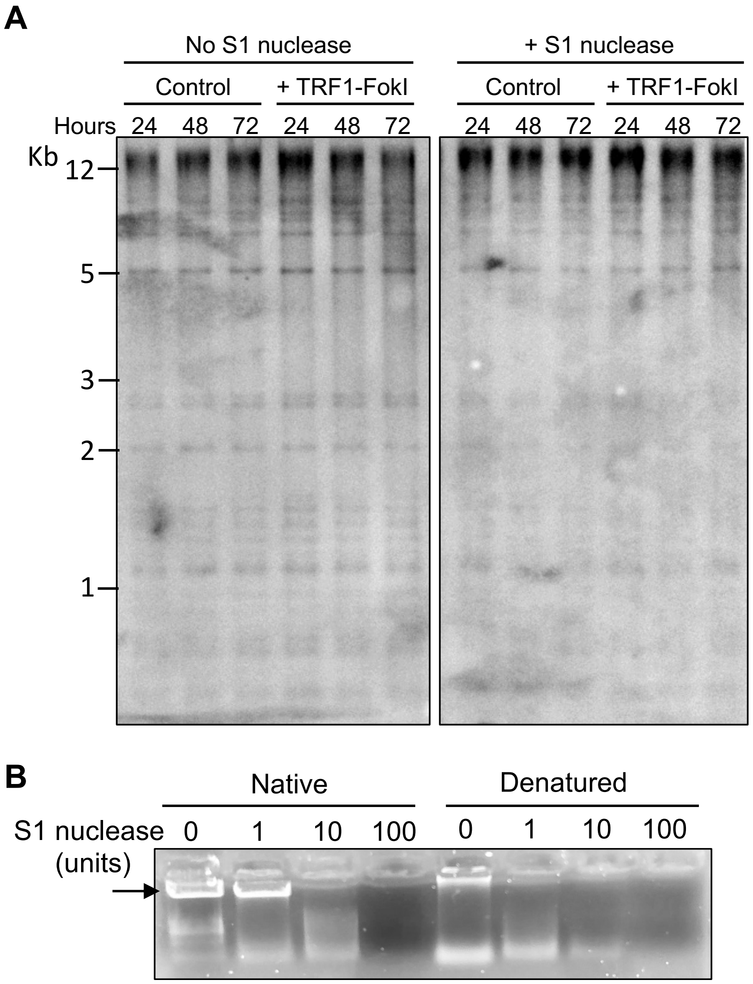

Figure S9: TRF1-FokI induction does not sensitize telomeres to digestion by S1 nuclease

A) TRF blot of genomic DNA treated with S1 nuclease. DNAs isolated from control iPSCs (treated with dox only) or iPSCs induced to express TRF1-FokI for the indicated times, were run without further treatment or after treatment with 1 unit of S1 nuclease. No apparent digestion with S1 nuclease was observed.

B) S1 nuclease titration under the same conditions as that used for the TRF blot. Native or heat-denatured genomic DNA (from the 72-hour control sample) was treated with the indicated amount of S1 nuclease and run on an ethidium bromide-stained gel. The mobility of genomic DNA is indicated by the *arrow*. Note that 1 unit of S1 nuclease was sufficient to degrade all of the denatured DNA (whereas $\geq$10 units degraded the native DNA as well).

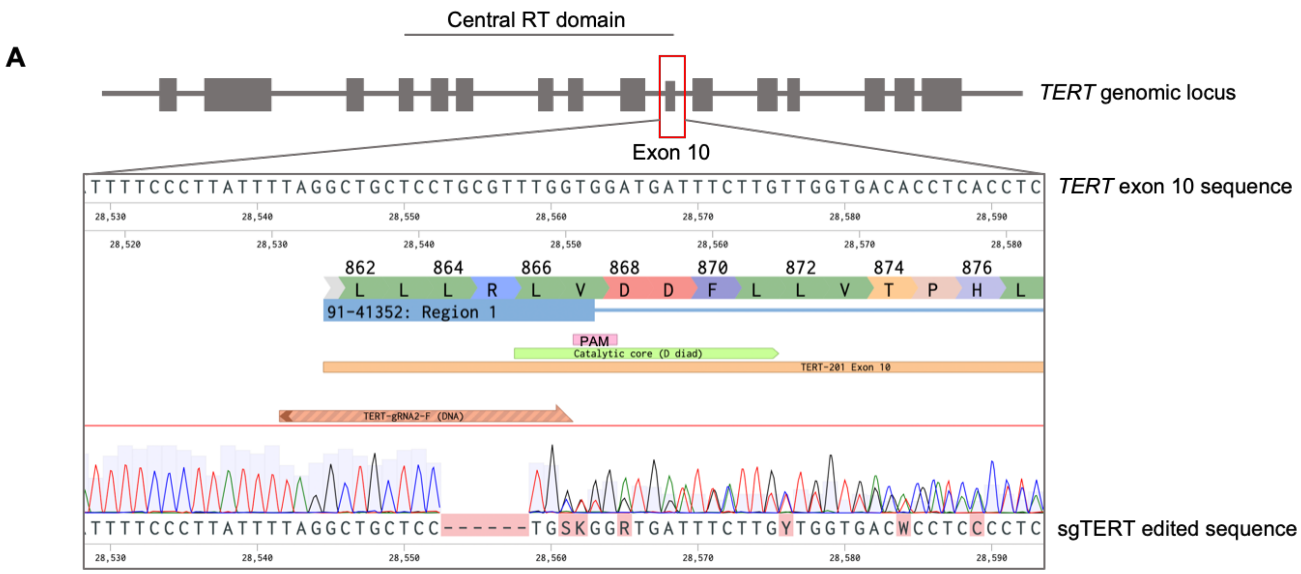

B

**Reference**	L	L	L	R	L	V	D	D	F	L	L	V	T	P	H	L	T	H	A	K	T	F	L	R	*
**Allele 1**	L	L	L	V	D	D	F	L	L	V	T	P	H	L	T	H	A	K	T	F	L	R	*		
**Allele 2**	L	L	L	R	G	G	*																		

Figure S10: Generation of biallelic *TERT* mutant TRF1-FokI iPSCs

A) CRISPR-based editing strategy for targeting exon 10 of the *TERT* locus in TRF1-FokI iPSCs and Sanger sequencing confirmation of an indel mutation adjacent to aspartate diads composing the catalytic core of the reverse transcriptase domain.

B) Amino acid sequence of resulting *TERT* alleles in CRISPR-targeted TRF1-FokI iPSCs.

Table S1: Key resources and reagents used in this study

Reagent Type (Species) or Resource	Designation	Source or Reference	Identifiers	Additional Information
**Cell Lines**				
iPS cell line (Homo sapiens)	PENN123i-SV20	(Pashos et al. 2017)		Parental
iPS cell line (Homo sapiens)	TRF1-FokI	This paper		Engineered
iPS cell line (Homo sapiens)	TRF1-FokI *TP53* KO	This paper		Engineered
iPS cell line (Homo sapiens)	TRF1-FokI sg*TERT*	This paper		Engineered
Fibroblast cell line (Homo sapiens)	IMR-90	(Nichols et al. 1977)		
ALT- control cell line (Homo sapiens)	HEK-293T	(Graham et al. 1977)		
ALT+ control cell line (Homo sapiens)	U2OS	(Ponten and Saksela 1964)		
**Recombinant DNA Reagents**				
Plasmids	pTRE-TIGHT-EGFP-donor	Paul Gadue	Addgene plasmid #22074	
Plasmids	AAVS1-SA-2A-NEO-CAG-RTTA3	Paul Gadue	Addgene plasmid #60431	
Plasmids	PGK-AAVS1ZFNR	Paul Gadue	Addgene plasmid #60915	
Plasmids	PGK-AAVS1ZFNL	Paul Gadue	Addgene plasmid #60916	
Plasmids	pLenti-CMV-Puro-DEST-TRF1-FokI	Roger Greenberg		
Plasmids	Px330		Addgene plasmid #42230	
Plasmids	pCAG-SpCas9-GFP-U6-gRNA		Addgene plasmid #79144	
Primer	AAVS1-WT-F	(Sim et al. 2016)		CCC CTA TGT CCA CTT CAG GA
Primer	AAVS1-WT-R	(Sim et al. 2016)		CAG CTC AGG TTC TGG GAG AG
Primer	AAVS1-CAG-F	(Sim et al. 2016)		GAG CAT CTG ACT TCT GGC TAA TA
Primer	AAVS1-CAG-R	(Sim et al. 2016)		GAA GGA TGC AGG ACG AGA AA
Primer	AAVS1-TRE-F	(Sim et al. 2016)		GCA ATA GCA TCA CAA ATT TCA C
Primer	AAVS1-TRE-R	(Sim et al. 2016)		GAA GGA TGC AGG ACG AGA AA (same as AAVS1-CAG-R)

Primer	Chr17 upstream F	This paper		AGA GAC GAG GTT TCA TCA TGT T
Primer	Chr17 upstream R	This paper		AAC AAC GTT CTG GTA AGG ACA A
Primer	Chr17 downstream F	This paper		TTT TCA GTT GTG CCA GCT TCA T
Primer	Chr17 downstream R	This paper		ACA GGA GAT GTT CTC ATA CAG GAG
Primer	TS	(Herbert et al. 2006)		AAT CCG TCG AGC AGA GTT
Primer	ACX	(Herbert et al. 2006)		GCG CGG CTT ACC CTT ACC CTT ACC CTA ACC
Primer	BranchedUniversal Primer	(Lai et al. 2016)		[Phos] GAC TCT CAA CTA TC+T +A
Primer	G-Rich ONT	(Lai et al. 2016)		[Phos] CCC TAA CCC TAA CCC TAA CCC TAA CCC TAA CCC TAA CCC TAA CCC TAG ATA GTT GAG AGT C
Primer	C-Rich ONT	This paper		
gRNA	p53 gRNA	This paper		AAT GAG GCC TTG GAA CTC A
gRNA	TERT gRNA	This paper		TAG GCT GCT CCT CGT TTG G
**Chemicals and Small Molecules**				
Chemical	Thiazovivin (TZV)	Cayman Chemical	CAT# 14245	
Chemical	Puromycin (hydochloride)	Cayman Chemical	CAT# 13884	
Chemical	G418 sulfate	Cayman Chemical	CAT# 13200	
Chemical	Doxycycline (dox)	Cayman Chemical	CAT# 14422	
Chemical	Shield-1 ligand	Aobious	CAT# AOB1848	
Chemical	4-OHT	Cayman Chemical	CAT# 17308	
Chemical	Nutlin-3	Cayman Chemical	CAT# 10004372	
Chemical	Ku-55933	Cayman Chemical	CAT# 16336	
Chemical	VE-821	Cayman Chemical	CAT #17587	
Chemical	CCT241533 (hydrochloride)	Cayman Chemical	CAT# 19178	
Chemical	LY2606368	Cayman Chemical	CAT# 21490	
Chemical	5-Bromo-2'-deoxyuridine	Cayman Chemical	CAT# 15580	
Chemical	5-Bromo-2'-deoxycytidine	Thermo Fisher	CAT# AAJ6545603	
Chemical	Hoechst	Thermo Fisher	CAT# 62249	
Chemical	DAPI	Cayman Chemical	CAT# 14285	
Chemical	Propidium iodide	Invitrogen	CAT# BMS500PI	
Chemical	Demecolcine solution	Sigma	CAT# D1925	
Chemical	TriZOL	Invitrogen	CAT# 15596018	
Chemical	Digoxigenin-11-dUTP	Sigma	CAT# 11093088910	

**Commercial Medias and Kits**				
Commercial Protein Product	Matrigel, Growth Factor Reduced	Corning	CAT# 354230	Can use with or without phenol red
Commercial Media	StemMACS iPS-Brew XF	Miltenyi Biotec	CAT# 130-104-368	
Commercial Media	mTeSR 1	StemCell Technologies	CAT# 85850	
Commercial Media	StemMACS passaging solution XF	Miltenyi Biotec	CAT# 130-104-688	
Enzymatic Reagent	Accutase	Innovative Cell Technologies	CAT# AT104-500	
Commercial Kit	CellEvent™ Senescence Green Flow Cytometry Assay Kit	Fisher Scientific	CAT# C10840	
Commercial Kit	CellTiter-Blue® Cell Viability Assay	Promega	CAT# G8081	
Commercial Kit	Lipofectamine™ Stem Transfection Reagent	Invitrogen	CAT# STEM00015	
Commercial Kit	Gentra Puregene Cell Kit	Qiagen	CAT# 158745	
Commercial Kit	Monarch RNA Cleanup Kit	NEB	CAT# T2040L	
Commercial Kit	Click-iT™ EdU Imaging Kit	Invitrogen	CAT# C10086	
**Antibodies and In-Situ Probes**				
Antibody	NANOG	Reprocell/Stemgent	CAT# 09-0020	1:250 4˚C overnight
Antibody	53BP1	Novus	CAT# NB100-304	1:250 4˚C overnight
Antibody	**γ**-H2AX	Millipore	CAT# 05-636	1:500 37˚C 1 hour
Antibody	Cleaved Caspase 3 (CC3)	Cell Signaling Technologies	CAT# 9664	1:1000 4˚C overnight
Antibody	Phospho-ATM (S1981)	R&D Systems	CAT# MAB22902-SP	1:1000 4˚C overnight
Antibody	Total ATM	Cell Signaling Technologies	CAT# 2873T	1:1000 4˚C overnight
Antibody	Phospho-ATR (Thr1989)	GeneTex	CAT# GTX128145	1:500 4˚C overnight
Antibody	Total ATR	ProteinTech	CAT# 19787-1-AP	1:500 4˚C overnight
Antibody	Chk1 (FI-476)	Santa Cruz	CAT# sc-7898	1:1000 4˚C overnight
Antibody	Chk2	Santa Cruz	CAT# sc-9064	1:1000 4˚C overnight
Antibody	TRF1	Millipore	CAT# 04-638	1:500 4˚C overnight
Antibody	TRF1	ProteinTech	CAT# 67592-1-Ig	1:500 4˚C overnight
Antibody	Estrogen receptor alpha (ERa)	Abcam	CAT# ab16660	1:500 4˚C overnight
Antibody	GAPDH	Abcam	CAT# ab9485	1:15000 4˚C overnight
Antibody	Phospho-Histone H3 (AF488 conjugated)	Cell Signaling Tehcnologies	CAT# 3465S	1:50 25˚C 90 min
Antibody	FITC Annexin V	BD Pharmingen	CAT# 560931	1:30 25˚C 30 min
Antibody	p53	ProteinTech	CAT# 10442-1-AP	1:1000 4˚C overnight

Antibody	PML	Santa Cruz	CAT# sc-966	1:1000 4˚C overnight
PNA Probe	Cy3-Telo-C Probe	PNA Bio	CAT# F1002	0.5 µg/ml
PNA Probe	488-Telo-G Probe	PNA Bio	CAT# F1008	0.5 µg/ml
Fab Fragments	Anti-Digoxigenin-AP, Fab fragments	Roche	CAT# 11093274910	1:20000 25˚C 30 min
Secondary Antibody	AlexaFluor 488 goat anti-rabbit IgG	Invitrogen	CAT# A-11034	1:500 25˚C 1 hour
Secondary Antibody	AlexaFluor 555 donkey anti-mouse IgG	Invitrogen	CAT# A-32773	1:500 25˚C 1 hour
Secondary Antibody	Rabbit anti-mouse HRP-conjugate	Abcam	CAT# ab97046	1:2000 25˚C 1 hour
Secondary Antibody	Goat anti-rabbit HRP-conjugate	Biorad	CAT# 170-6515	1:2000 25˚C 1 hour
**Molecular Biology Reagents**				
Restriction Enzyme	CviAII	NEB	CAT# R0640L	
Restriction Enzyme	BfaI	NEB	CAT# R0568L	
Restriction Enzyme	MseI	NEB	CAT# R0525L	
Restriction Enzyme	NdeI	NEB	CAT# R0111L	
Recombinant Enzyme	rSAP	NEB	CAT# M0371L	
Recombinant Enzyme	T4 DNA ligase	NEB	CAT# M0202S	
Molecular Biology Reagent	CDP-Star	Roche	CAT# 11759051001	
Modified Nucleotide	DIG-11-dUTP	Roche	CAT# 11558706910	
Molecular Biology Reagent	DIG Easy Hybridization Granules	Roche	CAT# 11796895001	
Molecular Biology Reagent	Blocking Reagent	Roche	CAT# 11096176001	
Recombinant DNA Marker	DIG Labeld DNA Molecular Weight Marker II	Roche	CAT# 11218590910	
Molecular Biology Reagent	Hybond-XL	Cytiva/Amersham	CAT# RPN303S	
Recombinant Enzyme	Klenow Fragment	NEB	CAT# M0212S	
Recombinant Enzyme	Lambda Exonuclease	NEB	CAT# M0262S	
Recombinant DNA Polymerase	Go-TAQ Flexi	Promega	CAT# M8298	
Recombinant DNA Polymerase	Phi29 DNA polymerase	NEB	CAT# M0269	
Recombinant Enzyme	Exonuclease I	NEB	CAT# M0293S	

Recombinant Enzyme	Exonuclease III	Promega	CAT# M1815	
Recombinant Enzyme	RNase A	Qiagen	CAT# 19101	
Recombinant Enzyme	Terminal transferase	NEB	CAT# M0315S	
**Software**				
Image Analysis Software	FIJI	NIH	RRID:SCR_002285	https://imagej.net/Fiji
Software	Benchling			benchling.com
Software	GraphPad Prism 9.2			
Software	Salmon			
Software	GSEA			
Flow Cytometry Analysis Software	FlowJo	BD Biosciences	RRID:SCR_008520	
Image Analysis Software	ImageQuant TL 8.2	Cytiva	RRID:SCR_018374	
